# A Case Report: Gastric glomus tumor with RAD50 mutation and therapeutic advances

**DOI:** 10.3389/fonc.2026.1745685

**Published:** 2026-03-04

**Authors:** Di Zhang, Wei Zhang, Lun Zhang, Jing Chen, Zhiliang Jin

**Affiliations:** 1 Department of Oncology, Jingzhou Hospital Affiliated to Yangtze University, Jingzhou, China; 2 School of Basic Medical Sciences, Health Science Center, Yangtze University, Jingzhou, China

**Keywords:** case report, chemotherapy, diagnosis, gastric glomus tumor, genetic test, immunohistochemistry

## Abstract

Gastric glomus tumor (GGT) is a rare mesenchymal neoplasm of the stomach that accounts for only 1% of gastrointestinal tumors and originates from glomus cells within the gastric wall. The exact etiology remains unclear, and due to nonspecific clinical manifestations, definitive diagnosis relies on imaging, pathology, and immunohistochemistry. We report a case of a 60-year-old female with a two-year history of unexplained upper abdominal burning pain. After undergoing abdominal CT, gastroscopy, pathology, and immunohistochemistry analyses, she was diagnosed with gastric glomus tumor complicated by hepatic metastasis. The patient subsequently received chemotherapy, transcatheter arterial embolization(TAE) for hemostasis, anti-infection treatment, blood transfusion, and symptomatic and supportive care at our hospital. Ultimately, the patient’s overall survival was 21 months. This article presents the case and reviews the clinical presentation, pathological features, diagnosis, and treatment strategies for GGT to provide reference for clinical practice.

## Introduction

Glomus tumors (GTs) are predominantly benign and originate from the glomus body, a thermoregulatory specialized arteriovenous structure (1). They can develop in any part of the body, most commonly occur in the distal extremities (2), particularly the subungual regions, palms, wrists, and feet—but can also appear in uncommon sites such as the nasal cavity, trachea, lungs, gastrointestinal tract, and peritoneum (3, 4). It is noteworthy that glomus tumors occurring in the retroperitoneum are often referred to as paragangliomas.

Gastric glomus tumors (GGTs) are exceedingly rare, first reported by De Busscher in 1948 (5) and only slightly over 300 cases of GGT have been reported to date. GGT is more common in women, with a male-to-female ratio of approximately 1:1.6 (6). A comprehensive overview of previously reported cases, including patient demographics, disease characteristics, treatment modalities, and outcomes, is provided in **Supplementary Material (Table 1)**. The median age of patients is 45 years, and tumors are mostly located in the gastric antrum (3, 7). Clinical manifestations are often nonspecific. The most common symptoms include abdominal pain or discomfort, anorexia, gastrointestinal bleeding, and ulcers, which may be accompanied by nausea and vomiting, frequently leading to misdiagnosis. Some are incidentally discovered during exams (8), and larger lesions may present with chronic ulceration and iron-deficiency anemia (9). Contrast-enhanced CT typically shows GGTs as well-defined, round or oval submucosal masses with strong arterial and persistent delayed enhancement (10). Endoscopic ultrasound (EUS) often demonstrates hypoechoic or mildly hyperechoic, hypervascular lesions with clear margins (11). However, due to similarities with other gastric submucosal tumors such as GISTs, leiomyomas, or neurogenic tumors, we believe that clinical symptoms and preoperative imaging are insufficient for a definitive diagnosis of GGT, definitive diagnosis requires histopathology and immunohistochemistry (12).

GGTs are well-demarcated but unencapsulated, composed of uniform round to polygonal cells with central nuclei arranged in perivascular, nested, or sheet-like patterns within a vascular stroma (13, 14). Immunohistochemically, they are positive for SMA, vimentin, calponin, and h-caldesmon (13), but negative for CD117, CD34, CK, CgA, desmin, S-100, CD56, and DOG-1, which helps distinguish them from other subepithelial tumors (11).

Although most GGTs are benign, malignant cases have been reported (7, 14–16). The first case of malignant GGT was reported by Haque (17)et al. in 1992. Malignant GGTs tend to recur and metastasize, with some cases demonstrating metastases to sites such as the liver, kidneys, and brain (14, 18). Folpe et al. (15) proposed malignant criteria, including deep location with a size >2 cm, high mitotic activity (≥5 mitoses per 50 HPFs), and moderate to severe nuclear atypia. The treatment approach varies depending on the nature and stage of GGT, with complete surgical resection remaining the primary treatment modality (19, 20). For small or benign tumors, wedge gastrectomy (21, 22) or endoscopic resection (23, 24) is typically employed. Minimally invasive surgery is often the preferred option due to its lower complication rates, faster recovery, and better patient quality of life (25, 26). In contrast, malignant GGTs are aggressive and prone to recurrence and metastasis. For larger or malignant tumors, subtotal or total gastrectomy may be required (18). However, depending on the patient’s condition, surgery might not be the first choice, and neoadjuvant or adjuvant therapies, including chemotherapy or radiotherapy, can be considered. Notably, lymph node metastasis is rarely reported in malignant GGT, so wide local excision with negative margins is usually sufficient, and lymphadenectomy is often unnecessary (27). Nevertheless, cases of metastasis via lymphatic and hematogenous routes have been documented (28, 29). For patients with extensive metastasis, the prognosis is generally poor and may ultimately lead to death. Given the rarity of the disease and the lack of a standard treatment protocol (21), multicenter studies are still needed to optimize therapeutic strategies.

## Case presentation

**Figure 1:**
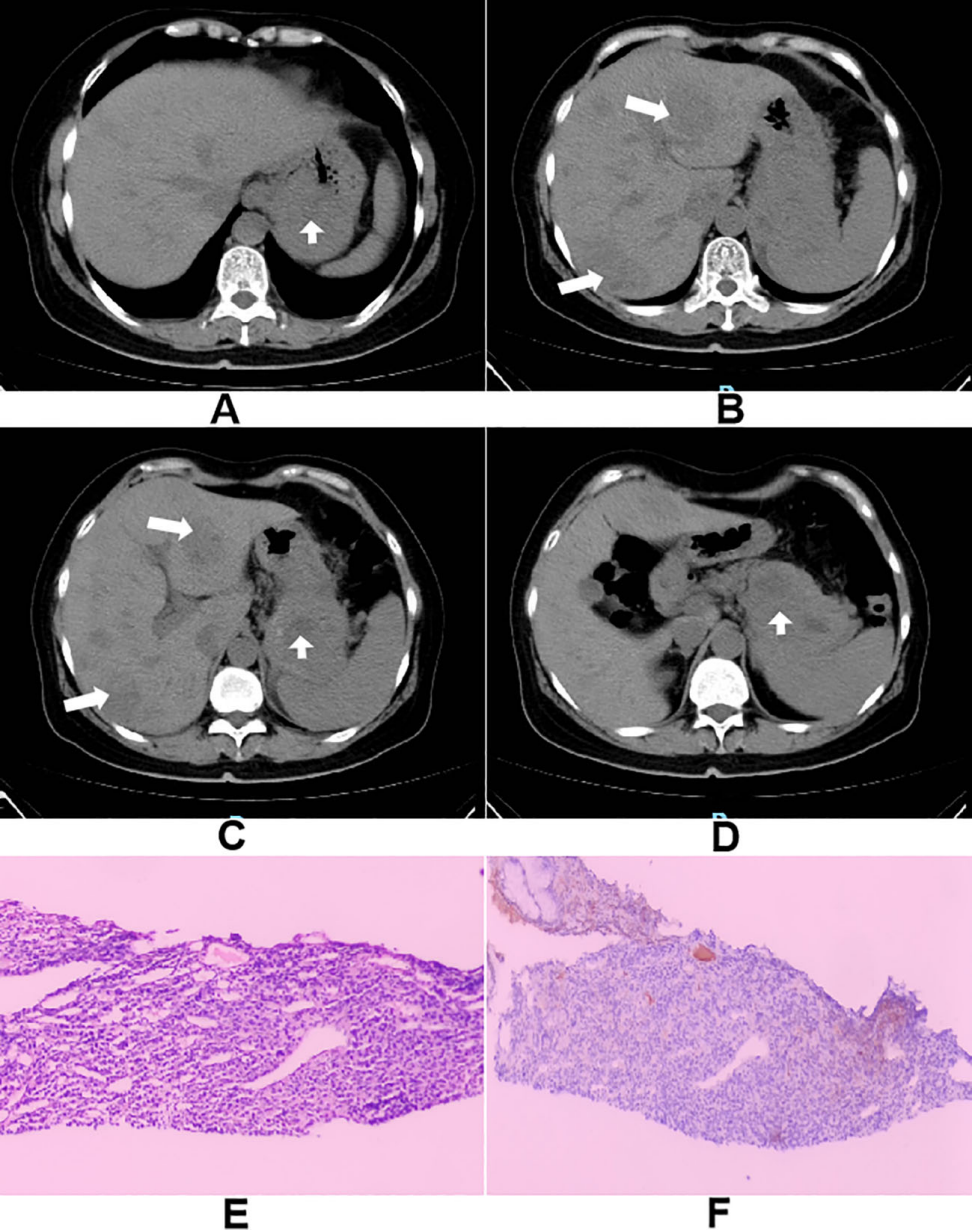
**(A–D)** Abdominal computed tomography showing an irregularly contoured gastric mass, with features of penetrating ulceration and multiple hepatic metastases; **(E)** Hepatobiliary metastatic malignant tumor immunohistochemical HE staining image; **(F)** PD-L1 protein expression detection image.

Here, we present a case of a 60-year-old female who presented with a one-month history of burning epigastric pain. Her symptoms later progressed to intermittent melena and high-grade fever (up to 39 °C). These symptoms severely affected her daily life and raised deep concerns about potential malignant disease, prompting further medical attention. After admission, a preliminary medical history was obtained: the patient was previously in good health. She underwent tonsil surgery 20 years ago. She denied a history of chronic diseases such as hypertension, diabetes, hepatitis, or tuberculosis, as well as any infectious diseases. There was no history of long-term medication use, smoking, or alcohol consumption. There is no family history of malignancy or hereditary diseases. Her psychosocial status was stable prior to the onset of illness, with no major life stressors. Physical examination revealed: temperature 38.9 °C, anemic appearance, and pale conjunctiva. Cardiopulmonary examination showed no significant abnormalities. The abdomen was flat and soft, with mild tenderness palpable below the xiphoid process in the epigastric region. No definite mass was felt, the liver and spleen were not palpable below the costal margin, and shifting dullness was negative. Digital rectal examination revealed melena. Abdominal CT revealed an irregularly gastric mass, suggestive of advanced gastric carcinoma with penetrating ulceration and multiple liver metastases (**Figures 1A–D**).To clarify the pathology, gastroscopic and biopsy was performed, confirming a malignant gastric mesenchymal tumor. However, precise classification was difficult because the morphology of the tumor in this location required differentiation from gastrointestinal stromal tumor (GIST), leiomyosarcoma, neuroendocrine tumor, etc., leading to diagnostic uncertainty. Further pathological and immunohistochemical results from an authoritative institution (Cancer Hospital, Chinese Academy of Medical Sciences) were consistent with features of a mesenchymal tumor, and glomus tumor could not be excluded. Subsequent liver biopsy and immunohistochemistry confirmed the diagnosis of malignant gastric glomus tumor with liver metastasis (stage IV) (**Figure 1E**). This disease is rare in advanced stages, highly aggressive, lacks standardized treatment, and carries a poor prognosis. The patient initially received empirical symptomatic management including anti−infection therapy (piperacillin−tazobactam), transfusion of packed red blood cells, acid suppression, and nutritional support. The high fever improved in the short term, but melena and anemia showed little improvement. This case report has been reported in line with the CARE guidelines and submit a completed CARE checklist as a **Supplementary Material (Table 2)**.

**Figure 2:**
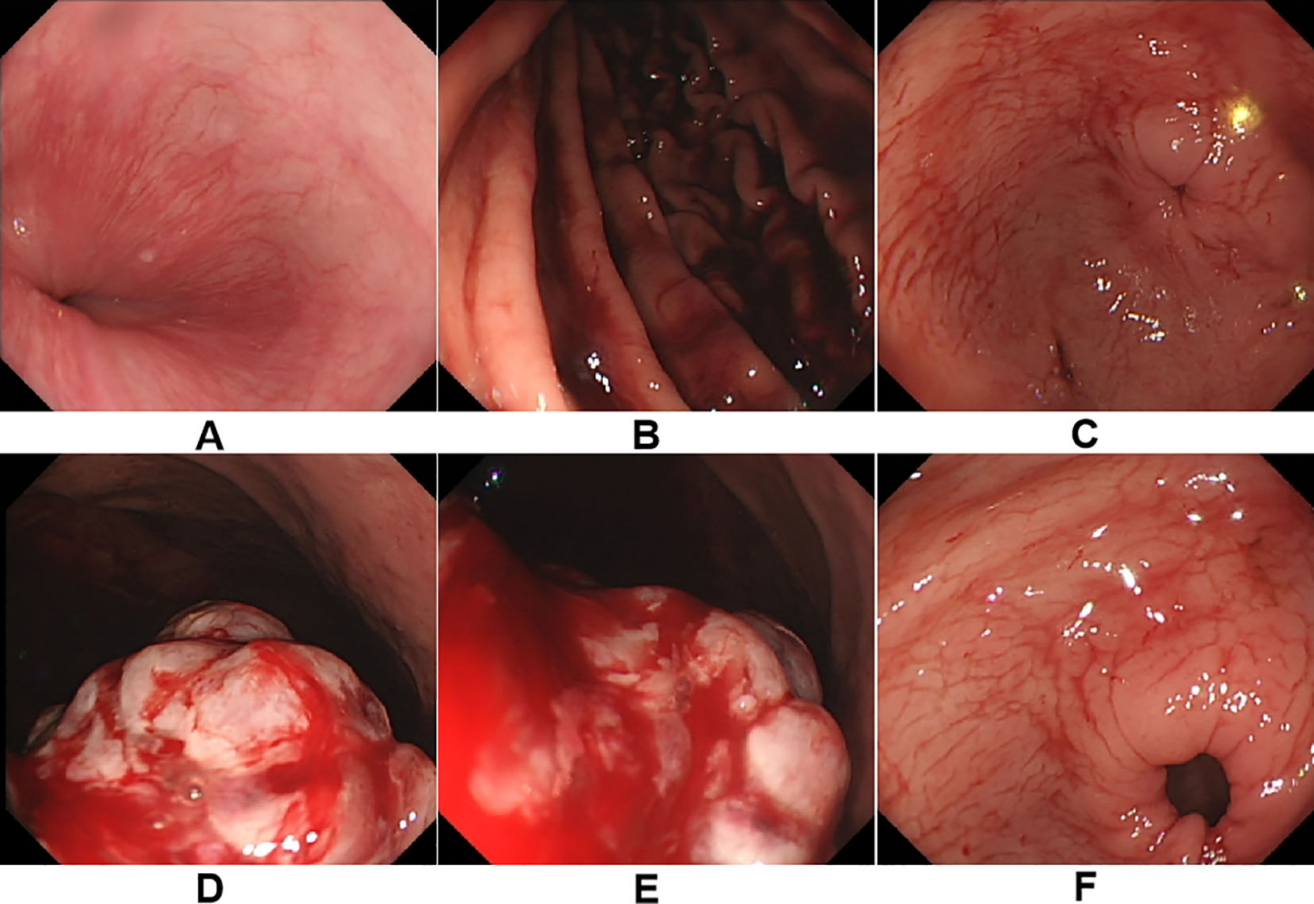
**(A–F)** A huge irregular mass can be seen at the cardia and fundus of the stomach, with surface ulceration and bleeding.

Two months later, the patient was admitted to our hospital for comprehensive oncologic management. Laboratory investigations revealed profound anemia (hemoglobin 49 g/L), but the levels of routine gastrointestinal tumor markers, such as carcinoembryonic antigen (CEA), carbohydrate antigen 19-9 (CA19-9), and alpha-fetoprotein (AFP), were all within normal limits. Repeat CT showed progression of gastric wall thickening, multiple liver metastases, and enlarged regional lymph nodes. Gastroscopy revealed a large, irregular ulcerated mass near the gastric cardia with visible ulceration and active bleeding (**Figures 2A–F**).

Given the rapidly progressive nature of the disease, the patient’s physical condition, and the ongoing risk of bleeding, along with the absence of a standard treatment protocol, empirical chemotherapy with albumin-bound paclitaxel monotherapy was selected following multidisciplinary team (MDT) discussion, based on its relatively rapid onset of action and potentially superior tolerability.

**Figure 3:**
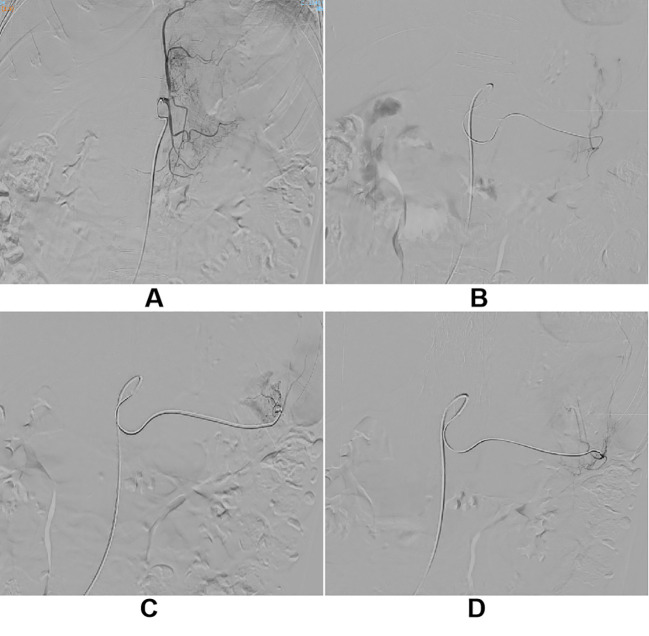
**(A–D)** The patient, presenting with persistent melena and anemia, underwent transcatheter gastric artery embolization under local anesthesia after a thorough medical evaluation.

**Figure 4:**
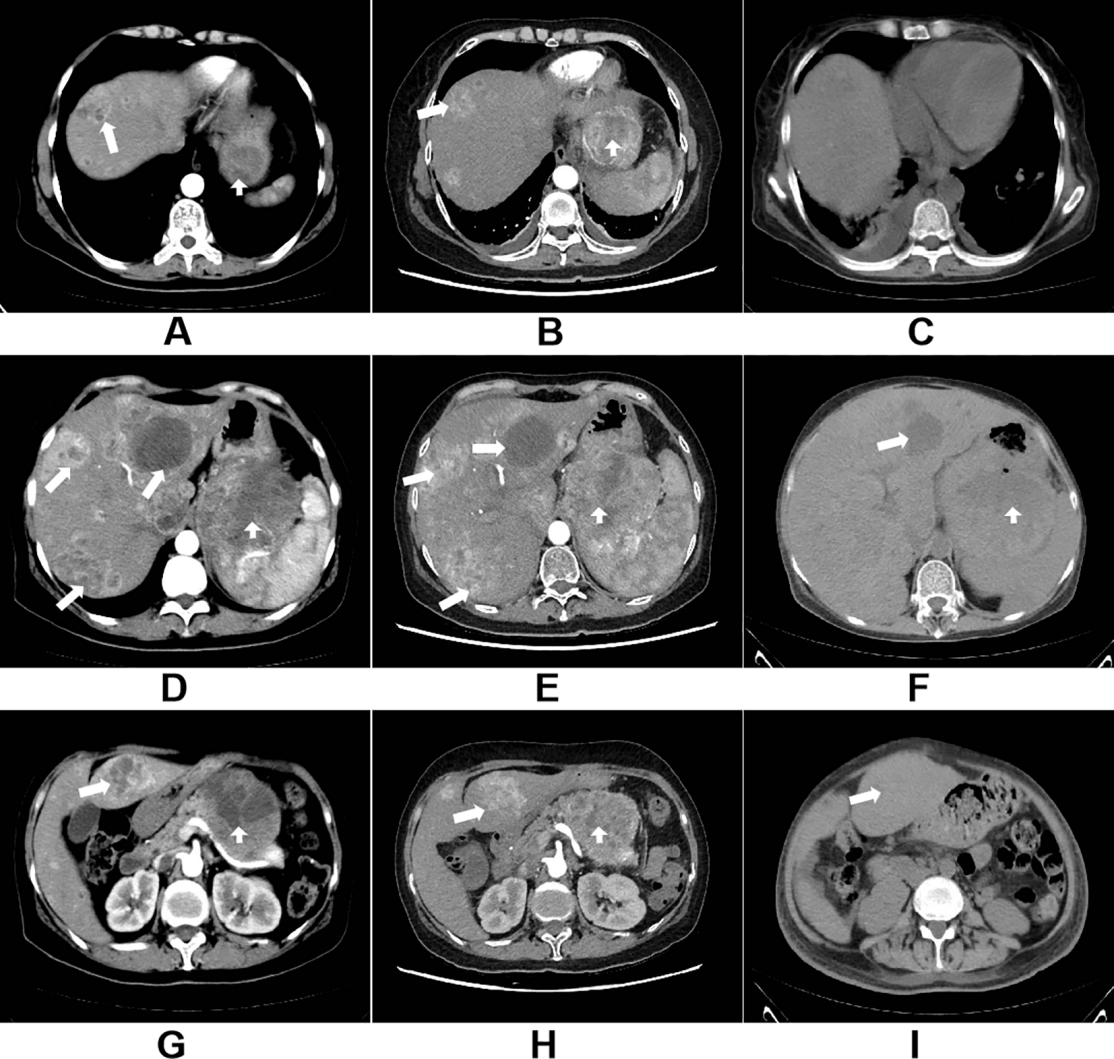
**(A, D, G)** The contrast-enhanced CT scan of the thorax and abdomen in January 2024 clearly shows the primary gastric hemangiopericytoma lesion and hepatic metastasis; **(B, E, H)** The enhanced CT scan of the thorax and abdomen in October 2024 shows marked disease progression, with enlargement of both the primary lesion and metastases, as well as the development of pleural effusion; **(C, F, I)** The non-contrast CT scan of the chest and abdomen in March 2025 indicates continuing disease progression.

The patient subsequently received one cycle of albumin-bound paclitaxel chemotherapy. However, gastrointestinal bleeding and anemia persisted, HB 40g/L, requiring ongoing transfusional support. To control the hemorrhage, transcatheter arterial embolization (TAE) of the left gastric artery was subsequently performed (**Figures 3A–D**). Post-procedure bleeding subsided. Imaging assessment according to RECIST 1.1 criteria indicated stable disease (SD). However, re-examination two months later revealed definitive progression of the liver metastases (**Figures 4A–I**), accompanied by recurrent low-grade fever and melena, marking the failure of first-line chemotherapy.

To explore further therapeutic options, comprehensive genetic testing was performed. It identified a pathogenic frameshift mutation in the RAD50 gene (exon 13, c.2165delA, p.K722fs), a key component of the homologous recombination repair pathway. Additional results showed microsatellite stability (MSS) and negative PD-L1 expression (CPS = 0) (**Figure 1F**)—suggesting limited benefit from immunotherapy—and several other somatic mutations including: STK11 copy number amplification, AR (exon 1: c.225_239dupGCAGCAGCAGCAGCA, p.Q76_Q80dup), ATM (exon 7: c.743G>A, p.R248Q), CCND2 (exon 4: c.718G>A, p.V240M), MRE11 (exon 20: c.2083_2085delGAT, p.D695del), PTCH1 (exon 3: c.431G>T, p.R144L). Based on the RAD50 mutation, targeted therapy with olaparib, a poly (ADP-ribose) polymerase (PARP) inhibitor was initiated but discontinued due to the patient’s severe vomiting following the first dose. A subsequent attempt with the anti-angiogenic tyrosine kinase inhibitor (TKI) lenvatinib was also terminated shortly after initiation owing to similar gastrointestinal toxicity. This highlighted the challenges in translating a theoretically “actionable target” into clinical benefit, especially against the backdrop of the patient’s compromised performance status and organ function.

Following a brief interruption in systemic therapy, imaging revealed further tumor progression, prompting a change to a second-line regimen of cyclophosphamide combined with liposomal doxorubicin. The patient completed 12 cycles over approximately 11 months with maintained radiographic stability. During this period, regular blood transfusions were required due to persistent melena. The patient then experienced massive hematemesis with hypovolemic shock, HB 28g/L, which was emergently managed with transfusions and a second TAE, achieving temporary stabilization. After recovery, third-line chemotherapy regimen consisting of 5-fluorouracil combined with liposomal irinotecan was administered over 4 cycles over about four months. Imaging continued to show stable disease, but the patient developed recurrent high fever (up to 39 °C), persistent melena, and remained transfusion-dependent. Her performance status had deteriorated to the point where she could no longer tolerate further chemotherapy. In an effort to suppress tumor angiogenesis and delay progression, recombinant human endostatin was administered for two cycles. Unfortunately, despite ongoing multimodal therapy, her hemoglobin levels declined rapidly, necessitating weekly transfusions of 3–6 units of packed red blood cells, with levels consistently between 60–70 g/L.

**Figure 5:**
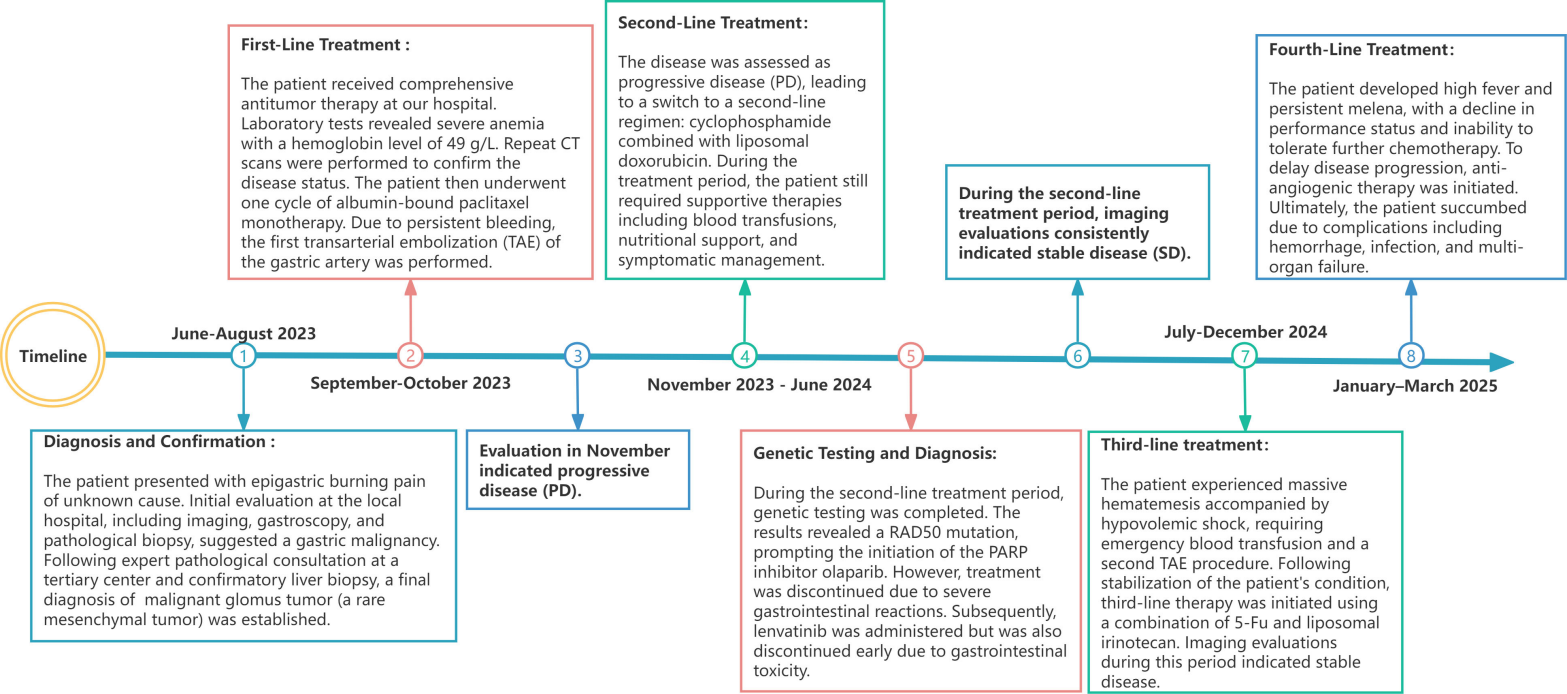
Timeline of diagnosis and treatment.

Throughout the treatment course, the patient received comprehensive supportive care including infection control, anemia management, fluid and electrolyte balance, and nutritional support. Ultimately, the patient succumbed to complications including uncontrolled gastrointestinal hemorrhage, high fever, sepsis, and multi-organ failure, with a total survival period of 21 months. All treatment modifications were based on radiographic progression, symptom exacerbation, declining tolerance, and limited evidence, and were preceded by full communication of the benefits and risks with the patient and family prior to each change. The timeline of this clinical case is summarized in **Figure 5**.

## Discussion

Gastric glomus tumors (GGTs) are rare gastric mesenchymal neoplasms (30), with malignant variants being exceedingly uncommon. Their nonspecific clinical and radiologic features—often resembling other submucosal tumors such as GISTs, leiomyomas, or neurogenic tumors—make accurate diagnosis challenging. Definitive identification typically requires a multidisciplinary approach integrating clinical evaluation, endoscopic findings, cross-sectional imaging, histopathological examination, and immunohistochemical profiling (12, 19).

In this case, initial diagnosis was based on endoscopy, imaging, and gastric biopsy findings suggestive of a malignant submucosal tumor. However, the final diagnosis of malignant GGT was confirmed only after immunohistochemical analysis of liver metastases. This underscores the diagnostic difficulty of GGTs and the importance of sampling metastatic lesions, particularly when primary biopsies are inconclusive.

Due to extensive hepatic metastases, surgical resection was not feasible at diagnosis. Furthermore, laboratory tests revealed severe anemia, and the patient was deemed not a suitable candidate for surgical intervention. The patient received multiple systemic therapies, yet only limited responses were observed, underscoring the potential chemoresistance of malignant GGTs. The rationale for selecting nab-paclitaxel monotherapy as the first-line treatment requires clarification here: due to the extreme rarity of malignant gastric glomus tumor (GGT), there is currently no standard protocol to guide treatment selection. Our decision was based on the following considerations: (1) Nab-paclitaxel is a broad-spectrum antineoplastic agent with activity against various malignancies of both epithelial and mesenchymal origin. The patient presented with persistent melena at initial diagnosis, which affected his performance status (PS) score. There was an urgent need to initiate systemic therapy promptly in an attempt to control the systemic disease. In the absence of a tumor-specific regimen for such a rare cancer, nab-paclitaxel is often considered an empirical option. (2) Compared to conventional paclitaxel formulations, nab-paclitaxel offers more convenient administration, a potentially lower risk of hypersensitivity reactions, and a possibly more favorable side effect profile, particularly regarding peripheral neurotoxicity. For a patient already weakened by tumor burden and chronic blood loss, these features were anticipated to improve treatment tolerance and help maintain quality of life. (3) Our hospital’s cancer center has extensive experience with the clinical application of this drug, facilitating dose adjustments and side-effect management. We acknowledge that this reflects the “trial-and-error” dilemma faced when treating ultra-rare tumors. The subsequent rapid progression confirmed the inefficacy of this regimen, prompting a switch to a second-line treatment. Notably, combined cyclophosphamide and liposomal doxorubicin maintained stable disease for 11 months, suggesting that some malignant GGTs may retain partial sensitivity to conventional soft tissue sarcoma regimens.

A major challenge in this case was recurrent gastrointestinal bleeding, manifesting as persistent melena, severe anemia, and repeated massive hemorrhages. These episodes required frequent blood transfusions and multiple transarterial embolization (TAE) procedures, indicative of the tumor’s hypervascularity and invasive behavior. Chronic bleeding not only compromised the patient’s quality of life but also reduced tolerance to systemic therapy, limiting treatment options. Given the patient’s recurrent active bleeding leading to anemia, we did consider the possibility of palliative tumor resection (such as proximal or total gastrectomy) to control hemorrhage. However, given that the patient already had multiple liver metastases (Stage IV) at the time of diagnosis, surgery could not achieve a cure. Furthermore, the patient’s overall condition was poor due to chronic blood loss and tumor burden. The gastric mass was large and located in a challenging area, meaning the surgical procedure would be highly invasive, carrying significant risks and a high likelihood of complications. After multidisciplinary team (MDT) discussion and thorough communication with the patient and family, it was concluded that prioritizing systemic therapy to control the overall disease, combined with local hemostatic measures such as endoscopy and interventional procedures, represented a strategy more aligned with the patient’s overall interests at that time. In this case, transcatheter arterial embolization (TAE) successfully achieved immediate hemostasis during two major bleeding episodes, demonstrating its utility in this context. This decision also highlights the need for careful balancing between disease control, patient quality of life, and treatment risks when making therapeutic decisions for advanced metastatic rare tumors.

Although anti-angiogenic therapies, including recombinant human endostatin and lenvatinib, were attempted to inhibit tumor angiogenesis and control bleeding, both were discontinued early due to either insufficient efficacy or intolerable gastrointestinal toxicity, resulting in no sustained clinical benefit. Molecular profiling revealed a pathogenic frameshift mutation in the RAD50 gene, a key component of the homologous recombination repair (HRR) pathway. While this finding suggested potential sensitivity to PARP inhibitors, the patient was unable to tolerate olaparib due to severe vomiting. Additionally, the tumor exhibited microsatellite stability (MSS) and PD-L1 CPS = 0, indicating a low likelihood of benefit from immune checkpoint inhibitors; hence, immunotherapy was not pursued.

This case illustrates several important considerations in managing malignant GGT. First, it highlights the potential disconnect between radiological disease stability and clinical deterioration—despite stable imaging, the patient experienced progressive symptoms and functional decline, emphasizing the need for symptom-guided, not solely imaging-based, treatment decisions. Second, it demonstrates the importance of genomic profiling in personalizing therapy, particularly for rare tumors lacking standard approaches. Here, next-generation sequencing (NGS) guided targeted treatment and enabled adaptive planning based on molecular traits, clinical status, lab trends, and imaging.

## Conclusion

This case report describes the challenges encountered in the diagnosis and treatment of an advanced metastatic gastric glomus tumor. In summary, this case highlights the significant difficulties in diagnosing and managing this rare malignancy. Although comprehensive multidisciplinary treatment provided the patient with a disease stabilization period exceeding 20 months, disease progression ultimately occurred. The next-generation sequencing (NGS) performed in this case revealed a potential RAD50 mutation, which theoretically suggests a possible benefit from immunotherapy or PARP inhibitors and provides a clue for future research. However, in the subsequent treatment of this patient, we were unable to validate these hypotheses due to factors such as bleeding risk and poor performance status. This profoundly illustrates the substantial gap that remains in translating genomic discoveries into tangible clinical benefits for ultra-rare tumors. While the NGS-guided personalized treatment attempt in this case did not yield significant clinical benefit, comprehensive molecular profiling still holds significant value. It helps confirm the diagnosis by ruling out other sarcomas with distinct molecular features and deepens our understanding of the biological behavior of such rare tumors. The management of advanced gastric glomus tumors still requires systematic accumulation of clinical and molecular data through international collaboration and multidisciplinary efforts in larger case series. Exploring ways to include such patients in basket or platform trials to establish treatment consensus is key to advancing therapy.

## Data Availability

The original contributions presented in the study are included in the article/**Supplementary Material**. Further inquiries can be directed to the corresponding author.
