# Supplementary material for: A Case Report: Gastric glomus tumor with RAD50 mutation and therapeutic advances
Source: Front Oncol. 2026 Mar 4;16:1745685. doi: 10.3389/fonc.2026.1745685 (PMC12995647; doi:10.3389/fonc.2026.1745685)
Supplement: Supplementary file 1 [file Table1.docx]

| **Case** | **Author, Year** | **Age** | **Sex** | **Past Medical History** | **Clinical Manifestations** | **Site** | **Size (cm)** | **EUS findings** | **CT findings** | **Immunohistochemical staining** | **Treatment** | **Prognosis** |
| --- | --- | --- | --- | --- | --- | --- | --- | --- | --- | --- | --- | --- |
| 1 | Song et al. 2025(1) | 60 | F | hypertension and hyperlipidemia | discovered incidentally during a physical exam | antrum | Gross examination : 3.0 × 2.0 × 1.0 cm | Esophagogastroduodenoscopy revealed a 10 mm dimple in the antrum | contrast-enhanced CT showed a 2.5 cm enhancing oval lesion | SMA(+),weak synaptophysin reactivity, and focal CD56 staining,CD117(-), HMB45(-), chromogranin A(-) | Laparoscopic partial gastrectomy with intraoperative endoscopic guidance | - |
| 2 | Yang et al. 2025(2) | elderly | M | healthy | discovered incidentally during a physical exam | antrum | EUS:28.8 ×22.5 mm Surgical: 30×30mm | the tumor originated from the muscular layer with minimal blood flow inside and detectable blood vessels behind it. Under the microscope, the tumor appeared to be composed of aggregated round and spindle-shaped cells surrounded by capillaries | a space-occupying lesion in the gastric antrum | SMA (+), vimentin (+); syn(-),C-kit(-) | laparoscopy combined with gastroscopy | The patient remained stable at the 1-year follow-up. |
| 3 | Nimura et al. 2025(3) | 35 | M | healthy | discovered incidentally during a physical exam | the greater curvature of the angulus | CT:1.7cm | a heterogeneous hyperechoic mass with dis tinct borders located in the proper muscle layer (fourth layer) of the stomach | a 17 mm mass in the stomach that showed homogeneous enhancement in the arte rial and portal phases and prolonged enhancement in the delayed phase | SMA (+)，cytokeratin AE1/AE3(-), synaptophysin(-), chromogranin A(-), DOG-1(-) | a laparoscopic partial gastrectomy without lymph node dissection. | Follow-up EGD at six months revealed no recurrence |
| 4 | Xu et al. 2025(4) | 36 | F | healthy | 4-day history of hematemesis, melena, and syncope | the side of the greater curvature of the stomach | CT:2cm | - | a 2cm lobulated gastric mass with heterogeneous enhancement and central necrosis | SMA (+), vimentin (+), pericellular type IV collagen(+), synaptophysin(+); Ki-67 was positive in up to 40% of tumor cells;CgA (-),CD56 (-),CK7 (-),CD117 (-),DOG-1 (-),CD34 (-),SSTR2A (-),LCA (-),CD99 (-),S-100 (-),epithelial membrane antigen (-) | wedge resection of the gastric lesion | A follow-up examination at 1 year postoperatively revealed liver metastases. Following treatment with radiofrequency ablation and laparoscopic resection of some lesions, the patient remained progression-free for 30 months during subsequent follow-up. |
| 5 | Toyomasu et al. 2025(5) | 55 | M | Helicobacter pylori gastritis | discovered incidentally during a physical exam | the anterior wall of the gastric antrum | CT: 3 cm  Surgical:3.5×3cm | a hypoechoic solid mass reaching the proper muscle layer. | the anterior wall of the gastric antrum thickened;seven months later,a 30 mm mass arising from the greater curvature of the stomach | SMA (+), vimentin (+)；c-kit(-), CD34(-), desmin(-), and S-100(-)； The MIB-1 labeling index was approximately 60 %. | laparoscopic partial gastrectomy | At the 2.5-year follow-up, liver metastases were identified. The patient continued to receive chemotherapy for 26 months and was in good general condition. |
| 6 | Fejes et al. 2024(6) | 52 | M | essential hypertension and five percutaneous coronary interventions | weakness and black stool for several days | in the middle third of the aboral side | Endoscopy: 40×30mm Surgical: 65×45 ×25mm | A repeated pan-gastroscopy revealed a 40 × 30 mm lacerated, polypoid lesion covered by mucosa, in the middle third of the aboral side, corresponding to the greater curvature, with multiple deep ulcerations but without active bleeding | a soft tissue mass bulging into the gastric lumen | SMA (+), CD34(+,only in endothelial cells),h-caldesmon(+,Strong but focal expression);the Ki-67 index was approximately 20% near the base of the ulcer and less than 10% elsewhere;CD117 (-) | laparoscopic sleeve resection | - |
| 7 | Thalji et al. 2024(7) | 34 | F | Hypothalamic hypogonadism | Upper GI bleeding | antrum | EUS:34×32 mm CT: 2.5×3.5×3 cm | Isoechoic mass was identified measuring about 34×32 mm with a well-defined border originating from the gastric muscularis propria layer | Soft tissue mass (white arrow) arising from the gastric antrum with heterogeneous enhancement in the arterial phase | SMA (+) | open laparotomy with antrectomy and Billroth II anastomosis | No recurrence at 2 years |
| 8 | Huang et al. 2024(8) | 49 | M | - | During a routine health check, gallstones were identified. Further investigation then revealed a mass in the gastric antrum. | antrum | Surgical: 5 cm × 4 cm × 3.5  cm | a hypoechoic mass in the intrinsic muscular layer of the gastric antrum | a mass-like high and low mixed density shadow in the anterior wall of the gastric antrum, with persistent heterogeneous enhancement in the arterial phase, and multiple stones in the gallbladder | SMA(+), vimentin(+), syn(+), H-caldesmon(+), and calponin(+);CgA(-), Dog-1(-), HMB-45(-), Desmin(-), S-100(-), CK-pan(-), and CD117(-);The proliferative index (Ki-67) in cellular areas was 20% | laparoscopic distal gastrectomy together with cholecystectomy | The patient remained stable over the 5-year follow-up period. |
| 9 | Bronze et al. 2024(9) | 59 | M | a medical history of arterial hypertension and dyslipidemia | 6-month history of dyspepsia, epigastric pain, and early satiety refractory to treatment with proton-pump inhibitor. | the distal gastric antrum contact with the pylorus | EUS: 3×2 cm  Surgical:3.5×3cm | a submucosal echogenic lesion measuring 30 × 20 mm with well-defined limits, without muscularis propria involvement. | - | SMA (+)，h-caldesmon (+) | submucosal tunneling endoscopic resection (STER) | At the 9-month follow-up visit, the patient was symptom-free. |
| 10 | Cláudio et al. 2024(10) | 56 | F | a history of chronic venous insufficiency | epigastric pain | the fundus-body transition | EUS：10.5 mm × 9.8 mm | an elevated, ulcerated, and irregular hypoechoic submucosal tumor (SMT), with a central anechoic area of probable origin in the fourth ultrasound layer, measuring 10.5 mm × 9.8 mm | a solitary gastric lesion (arrow) and a hypervascular enhancement on arterial phase. | SMA (+),type IV collagen(+),Ki-67 index of 2 %,KIT(-), CD34(-), desmin(-), chromogranin A(-), S100(-), synaptophysin(-) and DOG-1(-) | laparoscopic-endoscopic cooperative surgery | The patient is disease-free at 8-month follow-up with office visits every 4 months, asymptomatic and without late postoperative complications. |
| 11 | Deacu M et al, 2024 (11) | 65 | M | essential arterial hypertension, mitral insufficiency, ectasia of the ascending aorta, and New York Heart Association III heart failure stage C. To these were added chronic obstructive bronchopathy and dyspepsia. | Upper GI bleeding | Antrum | CT: 2.8×1.6 cm Macroscopically: 2.5×2×2 cm | - | a thickening of the gastric mucosa at the level of the submucosa of the antral region, an oval area with approximate dimensions of 28×16 mm. The contrast uptake was natively dense and heterogeneous | cytoplasmic and membrane collagen IV (+), smooth muscle acti (+), Ki-67 (+), vimentin (+). chromogranin A (-) DOG1 (-) | Surgical resection (gastrotomy, excision of the antral formation, and gastrorrhaphy) | - |
| 12 | Yu, F et al, 2023(12) | 52 | M | healthy | abdominal pain | antrum | EUS: 3cm | a mass about 3.0 cm in diameter in the gastric antrum with unclear muscle layer | - | vimentin(+), SMA(+), and Caldesmon(+)；CD34(-), DOG-1(-), S-100(-), CD34(-), and CD117(-), and the Ki-67 proliferation index was about 2%. | surgical treatment | - |
| 13 | Alkhateb, O et al, 2023 (13) | 45 | F | Approximately 6 months before the definitive diagnosis of gastric glomangiomyoma, she had dull, chronic left lower extremity pain and weakness. | severe epigastric left upper abdominal pain accompanied by melena, severe dizziness, as well as general malaise and fatigue. | Antrum | EUS: 4.0 × 2.4 cm Macroscopically:3 × 2 × 1.5 cm | hypoechoic homogenous lesion was seen to originate from the muscularis propria without invading the serosal layer, and occupied a quarter of the lumen | irregular hypertrophied mucosa at the antrum that extends into the pylorus. | CD34 (+), CD31(+), smooth muscle antigen (+), CD117 (-) | Surgical resection | - |
| 14 | Deng M et al, 2023(14) | 59 | F | - | A 10-year stomach-occupying lesion found on physical examination | Corpus | EUS: 1.5cm | - | - | collagen IV (+), (α-SMA) (+),vimentin (+),caldesmon-H (+), S100 (-),DOG1 (-), CD117 (-),desmin (-) | Subtotal gastrectomy | Loss-to- follow-up |
| 15 | Deng M et al, 2023(14) | 49 | F | - | Half-month recurrent hematemesis with black stool | Antrum | EUS: 2.7cm | - | - | collagen IV (+), (α-SMA) (+),vimentin (+),caldesmon-H (+), S100 (-),DOG1 (-), CD117 (-),desmin (-) | Radical gastrectomy | ANED, 63 months |
| 16 | Deng M et al, 2023(14) | 57 | F | - | Over 4 years of recurrent upper abdominal discomfort | Antrum | EUS: 2.5cm | - | - | collagen IV (+), (α-SMA) (+),vimentin (+),caldesmon-H (+), S100 (-),DOG1 (-), CD117 (-),desmin (-) | EFTR | ANED, 59 months |
| 17 | Deng M et al, 2023(14) | 65 | M | - | An 8-month gastric submucosal mass found on physical examination | Antrum | EUS: 1.2cm | - | - | collagen IV (+), (α-SMA) (+),vimentin (+),caldesmon-H (+), S100 (-),DOG1 (-), CD117 (-),desmin (-) | EFTR | ANED, 61 months |
| 18 | Deng M et al, 2023(14) | 49 | M | - | A 10-year stomach-occupying lesion found on physical examination | Corpus | EUS: 2.3cm | - | - | collagen IV (+), (α-SMA) (+),vimentin (+),caldesmon-H (+), S100 (-),DOG1 (-), CD117 (-),desmin (-) | Laparoscopic gastrectomy | Loss-to-follow-up |
| 19 | Deng M et al, 2023(14) | 35 | F | - | 2-year gastric submucosal protrusion on physical examination | Antrum | EUS: 1cm | - | - | collagen IV (+), (α-SMA) (+),vimentin (+),caldesmon-H (-), S100 (-),DOG1 (-), CD117 (-),desmin (-) | ESD | ANED, 57 months |
| 20 | Deng M et al, 2023(14) | 50 | M | - | 5-year progressive CEA elevation on physical examination | Corpus | EUS: 1cm | - | - | collagen IV (+), (α-SMA) (+),vimentin (+),caldesmon-H (-), S100 (+),DOG1 (+), CD117 (-),desmin (-) | ESD + subtotal gastrectomy + sentinel lymph-node dissection | ANED, 46 months |
| 21 | Deng M et al, 2023(14) | 62 | M | - | A 2-month gastric mass on physical examination | Antrum | EUS: 1.2cm | - | - | collagen IV (+), (α-SMA) (+),vimentin (+),caldesmon-H (+), S100 (-),DOG1 (+), CD117 (-),desmin (-) | EFTR | ANED, 37 months |
| 22 | Deng M et al, 2023(14) | 75 | F | - | A stomach-occupying lesion found on physical examination | Corpus | EUS: 4cm | - | - | collagen IV (+), (α-SMA) (+),vimentin (+),caldesmon-H (+), S100 (-),DOG1 (-), CD117 (-),desmin (-) | EFTR | ANED, 26 months |
| 23 | Deng M et al, 2023(14) | 58 | M | - | Intermittent abdominal pain with no predisposing factors, accompanying postprandial acid regurgitation for over half a year | Antrum | EUS: 1cm | - | - | collagen IV (+), (α-SMA) (+),vimentin (+),caldesmon-H (+), S100 (-),DOG1 (+), CD117 (-),desmin (-) | Subtotal antrectomy + subtotal duodenostomy | ANED, 27 months |
| 24 | Deng M et al, 2023(14) | 41 | F | - | Left upper abdominal pain for over 3 years and exacerbation for 4 years | Antrum | EUS: 2cm | - | - | collagen IV (+), (α-SMA) (+),vimentin (+),caldesmon-H (+), S100 (-),DOG1 (+), CD117 (-),desmin (-) | Laparoscopic gastrectomy | Loss-to-follow-up |
| 25 | Deng M et al, 2023(14) | 41 | F | - | A 4-month submucosal mass localized to the posterior wall of the antrum found on physical examination | Antrum | EUS: 1.5cm | - | - | collagen IV (+), (α-SMA) (-),vimentin (+),caldesmon-H (+), S100 (-),DOG1 (+), CD117 (-),desmin (-) | ESD | ANED, 16 months |
| 26 | Deng M et al, 2023(14) | 51 | F | - | 3-year submucosal protrusion of the antrum on physical examination | Antrum | EUS: 1cm | - | - | collagen IV (+), (α-SMA) (+),vimentin (+),caldesmon-H (+), S100 (-),DOG1 (-), CD117 (-),desmin (-) | ESD | ANED, 9 months |
| 27 | Deng M et al, 2023(14) | 36 | F | - | A stomach-occupying lesion found on physical examination | Corpus | EUS: 4cm | - | - | collagen IV (+), (α-SMA) (+),vimentin (+),caldesmon-H (-), S100 (-),DOG1 (-), CD117 (-),desmin (-) | Subtotal gastrectomy | ANED, 31 months; liver metastasis, 26 months postoperatively |
| 28 | Deng M et al, 2023(14) | 46 | F | - | Upper abdominal pain for over 2 months | Corpus | EUS:1.5cm | a 15 ⅹ 8 mm uniformed hypoechoic occupying lesion on ultrasound gastroscopy, with clear borders, originating from the submucosal layer with intact posterior mucosal layer | - | collagen IV (+), (α-SMA) (+),vimentin (+),caldesmon-H (+), S100 (-),DOG1 (-), CD117 (-),desmin (+) | ESD | ANED, 6 months |
| 29 | Malik, A et al, 2023(15) | 80 | M | hypertension and benign prostatic hypertrophy | Abdominal discomfort | Greater curvature | EUS: 1.56 x 1.28 cm | Subepithelial isoechoic, homogenous lesion with small calcifications on the gastric greater curvature abutting the left hepatic lobe | a 2.1 cm × 1.7 cm intraluminal mural mass projecting into the distal stomach lumen without obstruction. | neoplastic cells (+), smooth muscle actin (+), vimentin (+), patchy muscle-specific actin(+), and focal synaptophysin(+), pancytokeratin (-), desmin (-), CD117 (-), DOG1 (-), S100 (-), CK7 (-), CDX-2 (-), Pax-8 (-), HepPar1 (-), and CD34 (-) | Palliative (careful surveillance with serial abdominal imaging and EUS | - |
| 30 | Mohamed WT et al., 2023(16) | 27 | F | healthy | melena and lightheadedness | antrum | Surgical: 3.6 × 2.5 × 2.0 cm | - | 3-cm rounded prominence along the inferior wall of the gastric antrum adjacent to the metallic clip | SMA (+),calponin (+),pan-cytokeratin (-), chromogranin (-), synaptophysin (-), INSM1 (-), CD117 (-), CD34 (-), S100 (-), SOX10 (-), and pan melanoma markers (-). | laparoscopic robotic-assisted local wedge resection | On a follow-up visit 4 weeks later, she reported a complete resolution of symptoms. |
| 31 | Ezeh KJ et al., 2023 (17) | 67 | M | hypertension, hyperlipidemia, membranous urethral stricture status post direct vision internal urethrotomy, and cerebrovascular accident | weight loss and reflux | Body | Surgical: 1.5 x 1.0 x 0.6 cm EUS: 1.8 x 1.7 | an 18.5 x 17.5 mm lesion in the gastric wall from the muscularis propria, slightly hypoechoic with a heterogenous appearance, multiple vessels within the lesion, small area of cystic degeneration | mild thickening of the gastric wall without evidence of obstruction | synaptophysin (+), SMA(+), calponin (+), CD56 (-), chromogranin (-), Ki67~2% | Subtotal gastrectomy | - |
| 32 | Frosio F et al., 2023(18) | 60 | F | hypertension and Helicobacter pylori gastritis three years before,severe anemia | worsening asthenia and dyspnea on exertion. | Greater curvature | CT: 10cm | - | A mass arising from the greater curve of the stomach, which displayed contrast enhancement in the arterial and venous phases | Vimentin,(+) synaptophysin (+)., LCA (-), CD117 (-), EMA (-), SOX-1 (-), CK7 (-), MCK (-), DOG-1 (-), CK CAM 5.2 (-), and S100 (-) | Laparotomy: en-bloc wedge resection of the stomach and the transverse colon ;side-to-side double-layered hand-sewn colo-colonic anastomosis | Died |
| 33 | Ayash A et al, 2022 (19) | 61 | M | type 2 diabetes mellitus, hypertension, end‐stage renal disease on regular hemodialysis, coronary artery disease, and rectal cancer status post‐abdominoperineal resection with permanent colostomy. | Anemia and Melena | Antrum | CT:3cm | hyperechoic antral lesion originating from the second and third layer | well‐defined subepithelial soft tissue mass, bulging in the stomach lumen. The lesion showed heterogeneous post‐contrast enhancement. | smooth muscle actin (+), calponin (+), caldesmon (+), synaptophysin (+),CD 117 (-), CD 34 (-), CD 56 (-), S 100 (-), CK 20 (-), and chromogranin (-) | open wedge resection | The patient was doing well after 9 months of follow‐up post‐surgery. |
| 34 | Ezeh et al. 2022(20) | 50 | M | hypertension, prediabetes, and obesity | a one-month history of shortness of breath and fatigue worsening over the last one week before presenting | esser curvature | CT:3.7×2.5cm | a large submucosal mass measuring 15-20mm in the proximal part of the stomach on the lesser curvature | a 3.7x2.5cm calcified mass in the stomach | SMA(+), CD117(-), DOG-1(-), and CD34(-) | partial gastrectomy | - |
| 35 | Mehmood F et al, 2022(21) | 50 | F | obesity, hypertension, and coronary artery disease, and she was taking aspirin at home. | Dizziness and Hematemesis | Antrum | EGD :3cm | - | CT abdomen and pelvis with IV contrast showed no abdominal mass and scattered colonic diverticula without evidence of acute diverticulitis | (SMA) (+), pericellular collagen type IV (+),desmin (+), AE1/AE3 (-), S100 (-), inhibin (-), melan-A (-), and chromogranin (-). Synaptophysin, CD117, and STAT6 show non-specific background staining | Laparoscopic partial gastrectomy | She was doing well on follow-up visits. |
| 36 | Osama MA et al, 2022(22) | 60 | M | dyspepsia | Epigastric pain | Body | EUS: 2.2 x 1.8 cm | a hyperechoic submucosal lesion measuring 22 × 18 mm, suggestive of a lipoma. | - | SMA (+),synaptophysin (+),CD117 (-) and CD34 (-),Ki67 proliferative index was low < 1% | Distal gastrectomy with Roux-en-Y gastrojejunostomy | The patient is doing well and in a follow-up of 5 years; there has been no evidence of disease recurrence. |
| 37 | Bai B et al, 2021 (23) | 36 | F | - | Asymptomatic | Antrum | 1 cm | The fourth layer, round, hypoechoic, halos | well-defined subepithelial masses with homogeneous soft tissue densities with clear margins and perigastric adipose tissue | SMA (+), h-caldesmon (+),vimentin (+),CD117 (-), CD34 (-), S-100 (-), dog-1 (-), desmin (-), and CD31(-) | Endoscopic full-thickness resection | - |
| 38 | Bai B et al, 2021 (23) | 47 yr. | F | - | Asymptomatic | Lesser curvature | 1.2 cm | The fourth layer, round, hypoechoic, halos | well-defined subepithelial masses with homogeneous soft tissue densities with clear margins and perigastric adipose tissue | SMA (+), h-caldesmon (+),vimentin (+),CD117 (-), CD34 (-), S-100 (-), dog-1 (-), desmin (-), and CD31(-) | Endoscopic full-thickness resection | - |
| 39 | Bai B et al, 2021 (23) | 56 | F | - | Positive OBT | Lesser curvature | 3.5cm | The fourth layer, round, hypoechoic, halos | well-defined subepithelial masses with homogeneous soft tissue densities with clear margins and perigastric adipose tissue | SMA (+), h-caldesmon (+),vimentin (+),CD117 (-), CD34 (-), S-100 (-), dog-1 (-), desmin (-), and CD31(-) | Surgical resection | - |
| 40 | Bai B et al, 2021 (23) | 63 | M | - | Epigastric pain, heart burn | Antrum | 1.5 cm | The fourth layer, round, hypoechoic, halos | well-defined subepithelial masses with homogeneous soft tissue densities with clear margins and perigastric adipose tissue | SMA (+), h-caldesmon (+),vimentin (+),CD117 (-), CD34 (-), S-100 (-), dog-1 (-), desmin (-), and CD31(-) | Endoscopic full-thickness resection | - |
| 41 | Bai B et al, 2021 (23) | 65 | M | - | Epigastric pain, heart burn | Lesser curvature | 1.8cm | The fourth layer, round, hypoechoic, halos | well-defined subepithelial masses with homogeneous soft tissue densities with clear margins and perigastric adipose tissue | SMA (+), h-caldesmon (+),vimentin (+),CD117 (-), CD34 (-), S-100 (-), dog-1 (-), desmin (-), and CD31(-) | Endoscopic full-thickness resection | - |
| 42 | Bai B et al, 2021 (23) | 58 | M | - | heart burn | Lesser curvature | 2.3 cm | The fourth layer, round, hypoechoic, halos | well-defined subepithelial masses with homogeneous soft tissue densities with clear margins and perigastric adipose tissue | SMA (+), h-caldesmon (+),vimentin (+),CD117 (-), CD34 (-), S-100 (-), dog-1 (-), desmin (-), and CD31(-) | Endoscopic full-thickness resection | - |
| 43 | Bai B et al, 2021 (23) | 54 | F | - | heart burn | Lesser curvature | 2.1 cm | The fourth layer, round, hypoechoic, halos | well-defined subepithelial masses with homogeneous soft tissue densities with clear margins and perigastric adipose tissue | SMA (+), h-caldesmon (+),vimentin (+),CD117 (-), CD34 (-), S-100 (-), dog-1 (-), desmin (-), and CD31(-) | Endoscopic full-thickness resection | - |
| 44 | Bai B et al, 2021 (23) | 64 | M | - | Epigastric pain, heart burn | Lesser curvature | 2.2 cm | The fourth layer, round, hypoechoic, halos | well-defined subepithelial masses with homogeneous soft tissue densities with clear margins and perigastric adipose tissue | SMA (+), h-caldesmon (+),vimentin (+),CD117 (-), CD34 (-), S-100 (-), dog-1 (-), desmin (-), and CD31(-) | Endoscopic full-thickness resection | - |
| 45 | Bai B et al, 2021 (23) | 70 | M | - | Asymptomatic | Antrum | 1.3 cm | The fourth layer, round, hypoechoic, halos | well-defined subepithelial masses with homogeneous soft tissue densities with clear margins and perigastric adipose tissue | SMA (+), h-caldesmon (+),vimentin (+),CD117 (-), CD34 (-), S-100 (-), dog-1 (-), desmin (-), and CD31(-) | Endoscopic full-thickness resection | - |
| 46 | Bai B et al, 2021(23) | 56 | F | - | Epigastric pain, heart burn | Lesser curvature | 1.7 cm | The fourth layer, round, hypoechoic, halos | well-defined subepithelial masses with homogeneous soft tissue densities with clear margins and perigastric adipose tissue | SMA (+), h-caldesmon (+),vimentin (+),CD117 (-), CD34 (-), S-100 (-), dog-1 (-), desmin (-), and CD31(-) | Endoscopic full-thickness resection | - |
| 47 | Bai B et al, 2021 (23) | 55 | F | - | Positive OBT | Lesser curvature | 3.2 cm | The fourth layer, round, hypoechoic, halos, with anechoic areas | well-defined subepithelial masses with homogeneous soft tissue densities with clear margins and perigastric adipose tissue | SMA (+), h-caldesmon (+),vimentin (+),CD117 (-), CD34 (-), S-100 (-), dog-1 (-), desmin (-), and CD31(-) | Surgical resection | - |
| 48 | Bai B et al, 2021 (23) | 74 | F | - | Nausea | Lesser curvature | 2.8 cm | The fourth layer, round, hypoechoic, halos, with anechoic areas | well-defined subepithelial masses with homogeneous soft tissue densities with clear margins and perigastric adipose tissue | SMA (+), h-caldesmon (+),vimentin (+),CD117 (-), CD34 (-), S-100 (-), dog-1 (-), desmin (-), and CD31(-) | Surgical resection | - |
| 49 | Lee K et al, 2021(24) | 61 | M | thyroid papillary carcinoma on the left lobe of the thyroid gland underwent curative surgery (lobectomy with central node dissection) 3 years ago. | Incidental | Body | CT : 2.1 cm | hypoechoic tumor with homogeneous echogenicity and a well-circumscribed margin originating from the muscularis propria layer .The outer border of the tumor was irregular with a rubber-like consistency | well-enhancing nodule was noted in the gastric lower body | (α-SMA) (+), vimentin (+),cytokeratin AE1/AE (-). focal, weak positivity for CD56 and synaptophysin, | laparoscopic partial gastrectomy | The patient was evaluated at the first and sixth months post gastrectomy and at routine postoperative exams, including an esophagogastroduodenoscopy and enhanced abdominopelvic CT, which did not show any abnormal findings or residual tumor. |
| 50 | Brotherton T et al, 2021(25) | 44 | F | multiple sclerosis, chronic constipation, and depression;laparoscopic cholecystectomy | Abdominal pain | Greater curvature | CT: 2.2 x 1.8 x 2.0 cm | - | submucosal, mixed density, well-circumscribed mass | collagen IV (+), smooth muscle actin (+), vimentin (+)desmin (+). CD117 (-) , DOG-1 (-).,synaptophysin was weakly positive chromogranin (-), pancytokeratin (-),CAM5.2 (-), S100 (-) | Partial laparoscopic gastrectomy | - |
| 51 | Tantia M et al, 2021(26) | 28 | F | - | Abdominal pain and hematemesis | Antrum | CT: 3.7 x 3.3 cm | - | well-defined lesion in the antrum, | (SMA) (+), caldesmon-H (+) actin (+) , CD117 (-), DOG1 (-), CD34 (-), pan CK (-), synaptophysin (-) and chromogranin (-) | Laparoscopic resection | At 1 year of follow-up, the patient was asymptomatic. |
| 52 | Vyawahare, M et al, 2021(27) | 42 | F | - | Hematemesis and melena | Antrum | CT: 2.5 X 2.5 cm Macroscopic lly: 2.5 cm × 3 cm | - | contrast-enhanced focal well-defined 2.5 cm × 2.5 cm mass lesion in gastric antrum | SMA (+), Synaptophysin (+), Caldesmon (+), Ki67 (+),CD117 (-), S100 (-), Chromogranin (-) and Pan CK (-) | Laparotomy with wedge resection | - |
| 53 | W. Wang et al., 2021(28) | 52 | M | healthy | Epigastric discomfort and sense of fullness | Antrum | Surgical: 3×3  EUS: 2.4×1.8  CT: 3.0 × 2.2 | mass originated from the muscularis propria and exhibited both intraluminal and extraluminal growth, with hypoechoicity on the periphery, hyperechoicity in the middle, and unclear boundaries | Nodular thickening in the gastric wall and after enhancement, the lesion exhibited obvious enhancement | Spinal muscular atrophy (+), h-caldesmon (+), cluster of differentiation 34 (CD34) (+), 2% Ki-67-positive rate, CD56 (-), melanoma antigen (-), CD117 (-), leukocyte common antigen (-), caudal type homeobox 2 (-), cytokeratin (-), and S-100 (-) | Laparoscopy gastroscopic cooperative surgery | - |
| 54 | R. Mendo et al., 2021(29) | 37 | F | healthy | Epigastric pain and bloating | Antrum | EUS: 2.5 | Hypoechogenic subepithelial lesion originating in the muscularis propria | - | Smooth-muscle actin (+) ,synaptophysin (+), chromogranin (-), CD117 (-),KIT and PDGFRA mutations (-) | Laparoscopic wedge gastrectomy | - |
| 55 | S. Sethi et al., 2021(30) | 35 | F | - | Upper GI bleeding | Greater curvature | CT: 4x3 | - | Hypodense, polypoid lesion with enhancement in the arterial phase and persistent progressive enhancement in the portal venous phase | SMA (+), vimentin (+), caldesmon (+), synaptophysin (-), chromogranin (-), CD 34 (-), CD117 (-), Mib - 1 index was < 1%. | Antrectomy with Billiroth 1 reconstruction | - |
| 56 | A.G. Alsahwan et al., 2021(31) | 56 | M | healthy | Upper GI bleeding and signs of shock | Greater curvature | Surgical: 7×4×2.5 | - | A large lobulated mass involving the greater curvature, with no local invasion or distant metastasis | SMA (+), vimentin (+), calponin (+), synaptophysin (+), CK (AE1/AE3) (-), chromogranin A (-), desmin (-), CD117 (C-KIT) (-), Dog1(-) and S100 (-).The Ki67 index was around 30%. | Wedge gastrectomy | At follow-up, 3 and 6 months after the surgery, the patient had no complaints. |
| 57 | E.S. Tsagkataki et al., 2021(32) | 53 | M | healthy | Fatigue and black stool | Between the pylorus of the stomach and the first part of the duodenum | Surgical:  5.5×5×4.2 | - | Mass with vague limits, several lymph nodes with diameter up to 1.6 cm noticed around the mass | SMA (+), CD117 (c-kit) (-) and desmin (-) | Antrectomy with Roux-en-Y anastomosis and appendectomy | His last follow-up was in January 2021, 20 months after his admission to the hospital, in which he was found in good clinical status and with no signs of recurrence. |
| 58 | S. Singh et al., 2020(33) | 24 | F | healthy | mild-to-moderate pain abdomen in the right lumbar region | Antrum and pyloric canal | EUS: 3.8×2.5cm Surgical: 3.1×3.3×3.5cm | 3.8 cm ×2.5 cm-sized heteroechoic submucosal solid gastric mass in the antrum | a well-defined smoothly marginated soft-tissue density submucosal lesion in relation to the antrum and pyloric canal of the stomach, bulging in its lumen, as well as causing bulge of its external contour laterally | SMA (+), Ki67 (+), CK (-), synaptophysin (-), DOG-1(-), and desmin (-) | Distal gastrectomy with Roux-en-Y anastomosis | - |
| 59 | T. Hansen et al., 2020 (34) | 72 | M | - | Weight loss, diarrhea, and epigastric pain | Antrum | CT: 2.9×2.7 | A well-circumscribed tumor mass was found in the gastric wall | A well-circumscribed mass with no evidence of infiltration in the surrounding fat tissue. | SMS (+), cytokeratin (-), CD34 (-), CD117 (-), DOG‑1 (-), desmin (-), and CD45 (-) | Wedge gastrectomy | - |
| 60 | S. Mago et al., 2020(35) | 42 | M | hypertension and gastroesophageal reflux disease | Left upper quadrant pain and multiple episodes of  emesis | Incisura | CT: 3.2 x 2.7 x 3.1  EUS:3.3 | Well-defined hypoechoic lesion arising from the muscularis propria (EUS layer 4) | Soft tissue mass with central calcification and vascularity along the lesser curvature of the stomach | SMA (+), synaptophysin (+), along with weak staining for placental Alkaline phosphatase (PLAP), CD117 (-), CD34 (-), Desmin (-), S-100(-), DOG1 (-) and sparse Ki67 staining (-) | Surgical wedge resection | On six months follow up, the patient recovered well and symptoms resolved. |
| 61 | H. Hasuda et al, 2020(36) | 64 | M | diabetes and hypothyroidism | discovered incidentally during a physical exam | Lesser curvature | Surgical: 1.3×1.1×03 EUS: 1.2 | A hypoechoic tumor in the second and third layers of the stomach wall | Difficult to identify due to small tumor size and poor image enhancement | SMA (+), h-caldesmon (+), collagen type IV (+), desmin (-), S-100 protein (-), CD 34 (-), epithelial membrane antigen (-), and cytokeratin AE1/AE3 (-) | Laparoscopy and endoscopy cooperative surgery (LECS) | - |
| 62 | U.G. Rossi et al., 2020(37) | 49 | M | - | Intermittent increasing epigastric discomfort | Antrum | CT: 4 | - | An intraluminal subepithelial mass, well-defined, with high vascular enhancement | Cytokeratin (-) and CD56 (-) | Wedge gastrectomy | - |
| 63 | I.V. Branco et al., 2020(38) | 70 | M | - | Nausea, anorexia, dizziness, and melena | Antrum | CT:2.4 | - | A well-circumscribed, submucosal, nodular mass, with increasing contrast enhancement from arterial to venous phase | SMA (+), synaptophysin (+) and CD34 (+) | Partial gastrectomy with a gastro-jejunal Roux-en-Y anastomosis | - |
| 64 | J. Lin et al.,2020(39) | 59 | F | - | Epigastric discomfort | Antrum | Surgical: 3.5 | - | - | Vimentin (+), SMA (+) Partial expression of Hcaldesmon was observed. Focal or more extensive positivity for Syn was observed in three cases,  For cga (-) and NSE (-). AE1/AE3 (-), CD117 (-), LCA (-), S100 (-), and CD34 (-) | Segmental resection | - |
| 65 | J. Lin et al.,2020(39) | 51 | F | - | Epigastric discomfort | Antrum | Surgical: 1.5 |  | - | Vimentin (+), SMA (+) Partial expression of Hcaldesmon was observed. Focal or more extensive positivity for Syn was observed in three cases,  For cga (-) and NSE (-). AE1/AE3 (-), CD117 (-), LCA (-), S100 (-), and CD34 (-) | Segmental resection | - |
| 66 | J. Lin et al.,2020(39) | 44 | F | - | Epigastric discomfort | Antrum | Surgical: 2.5 | - | - | Vimentin (+), SMA (+) Partial expression of Hcaldesmon was observed. Focal or more extensive positivity for Syn was observed in three cases,  For cga (-) and NSE (-). AE1/AE3 (-), CD117 (-), LCA (-), S100 (-), and CD34 (-) | Segmental resection | - |
| 67 | J. Lin et al.,2020(39) | 62 | M | - | Epigastric pain | Antrum | Surgical: 1.5 | - | - | Vimentin (+), SMA (+) Partial expression of Hcaldesmon was observed. Focal or more extensive positivity for Syn was observed in three cases,  For cga (-) and NSE (-). AE1/AE3 (-), CD117 (-), LCA (-), S100 (-), and CD34 (-) | Segmental resection | - |
| 68 | J. Lin et al.,2020(39) | 40 | M | - | - | Antrum | Surgical: 2.3 | - | - | Vimentin (+), SMA (+) Partial expression of Hcaldesmon was observed. Focal or more extensive positivity for Syn was observed in three cases,  For cga (-) and NSE (-). AE1/AE3 (-), CD117 (-), LCA (-), S100 (-), and CD34 (-) | Segmental resection | - |
| 69 | J. Lin et al.,2020(39) | 25 | F | - | Melena | Antrum | Surgical: 2.6 | - | - | Vimentin (+), SMA (+) Partial expression of Hcaldesmon was observed. Focal or more extensive positivity for Syn was observed in three cases,  For cga (-) and NSE (-). AE1/AE3 (-), CD117 (-), LCA (-), S100 (-), and CD34 (-) | Segmental resection | - |
| 70 | J. Lin et al.,2020(39) | 46 | M | - | - | Antrum | Surgical: 2.5 | - | - | Vimentin (+), SMA (+) Partial expression of Hcaldesmon was observed. Focal or more extensive positivity for Syn was observed in three cases,  For cga (-) and NSE (-). AE1/AE3 (-), CD117 (-), LCA (-), S100 (-), and CD34 (-) | Segmental resection | - |
| 71 | J. Lin et al.,2020(39) | 43 | M | - | Epigastric discomfort | Antrum | Surgical: 2 | - | - | Vimentin (+), SMA (+) Partial expression of Hcaldesmon was observed. Focal or more extensive positivity for Syn was observed in three cases,  For cga (-) and NSE (-). AE1/AE3 (-), CD117 (-), LCA (-), S100 (-), and CD34 (-) | Segmental resection | - |
| 72 | J. Lin et al.,2020(39) | 54 | M | - | Epigastric discomfort | Antrum | Surgical: 2.3 | - | - | Vimentin (+), SMA (+) Partial expression of Hcaldesmon was observed. Focal or more extensive positivity for Syn was observed in three cases,  For cga (-) and NSE (-). AE1/AE3 (-), CD117 (-), LCA (-), S100 (-), and CD34 (-) | Segmental resection | - |
| 73 | J. Lin et al.,2020(39) | 40 | F | - | Epigastric discomfort | Antrum | Surgical: 2 | - | - | Vimentin (+), SMA (+) Partial expression of Hcaldesmon was observed. Focal or more extensive positivity for Syn was observed in three cases,  For cga (-) and NSE (-). AE1/AE3 (-), CD117 (-), LCA (-), S100 (-), and CD34 (-) | Segmental resection | - |
| 74 | J. Lin et al.,2020(39) | 34 | F | - | Epigastric pain | Antrum | Surgical: 2 | - | - | Vimentin (+), SMA (+) Partial expression of Hcaldesmon was observed. Focal or more extensive positivity for Syn was observed in three cases,  For cga (-) and NSE (-). AE1/AE3 (-), CD117 (-), LCA (-), S100 (-), and CD34 (-) | Endoscopic resection | - |
| 75 | J. Lin et al.,2020(39) | 37 | F | - | Epigastric discomfort | Body | Surgical: 1.5 | - | - | Vimentin (+), SMA (+) Partial expression of Hcaldesmon was observed. Focal or more extensive positivity for Syn was observed in three cases,  For cga (-) and NSE (-). AE1/AE3 (-), CD117 (-), LCA (-), S100 (-), and CD34 (-) | Subtotal gastrectomy | - |
| 76 | J. Lin et al.,2020(39) | 54 | M | - | Epigastric pain | Antrum | Surgical: 0.8 | - | - | Vimentin (+), SMA (+) Partial expression of Hcaldesmon was observed. Focal or more extensive positivity for Syn was observed in three cases,  For cga (-) and NSE (-). AE1/AE3 (-), CD117 (-), LCA (-), S100 (-), and CD34 (-) | Endoscopic resection | - |
| 77 | J. Lin et al.,2020(39) | 60 | M | - | Epigastric pain | Antrum | Surgical: 2.7 | - | - | Vimentin (+), SMA (+) Partial expression of Hcaldesmon was observed. Focal or more extensive positivity for Syn was observed in three cases,  For cga (-) and NSE (-). AE1/AE3 (-), CD117 (-), LCA (-), S100 (-), and CD34 (-) | Endoscopic resection | - |
| 78 | J. Lin et al.,2020(39) | 45 | M | - | Epigastric discomfort | Antrum | Surgical: 1.2 | - | - | Vimentin (+), SMA (+) Partial expression of Hcaldesmon was observed. Focal or more extensive positivity for Syn was observed in three cases,  For cga (-) and NSE (-). AE1/AE3 (-), CD117 (-), LCA (-), S100 (-), and CD34 (-) | Segmental resection | - |
| 79 | J. Lin et al.,2020(39) | 60 | M | - | Epigastric discomfort | Antrum | Surgical: 2.3 | - | - | Vimentin (+), SMA (+) Partial expression of Hcaldesmon was observed. Focal or more extensive positivity for Syn was observed in three cases,  For cga (-) and NSE (-). AE1/AE3 (-), CD117 (-), LCA (-), S100 (-), and CD34 (-) | Segmental resection | - |
| 80 | J. Lin et al.,2020(39) | 42 | F | - | Epigastric pain | Antrum | Surgical: 1.5 | - | - | Vimentin (+), SMA (+) Partial expression of Hcaldesmon was observed. Focal or more extensive positivity for Syn was observed in three cases,  For cga (-) and NSE (-). AE1/AE3 (-), CD117 (-), LCA (-), S100 (-), and CD34 (-) | Segmental resection | - |
| 81 | J. Lin et al.,2020(39) | 55 | M | - | Epigastric discomfort | Antrum | Surgical: 1.5 | - | - | Vimentin (+), SMA (+) Partial expression of Hcaldesmon was observed. Focal or more extensive positivity for Syn was observed in three cases,  For cga (-) and NSE (-). AE1/AE3 (-), CD117 (-), LCA (-), S100 (-), and CD34 (-) | Segmental resection | - |
| 82 | J. Lin et al.,2020(39) | 61 | F | - | Melena | Antrum | Surgical: 2.8 | - | - | Vimentin (+), SMA (+) Partial expression of Hcaldesmon was observed. Focal or more extensive positivity for Syn was observed in three cases,  For cga (-) and NSE (-). AE1/AE3 (-), CD117 (-), LCA (-), S100 (-), and CD34 (-) | Segmental resection | - |
| 83 | J. Lin et al.,2020(39) | 68 | F | - | Epigastric discomfort | Antrum | Surgical: 1.5 | - | - | Vimentin (+), SMA (+) Partial expression of Hcaldesmon was observed. Focal or more extensive positivity for Syn was observed in three cases,  For cga (-) and NSE (-). AE1/AE3 (-), CD117 (-), LCA (-), S100 (-), and CD34 (-) | Total gastrectomy | died 13 months after the operation. |
| 84 | J. Lin et al.,2020(39) | 55 | F | - | - | Antrum | Surgical: 2.7 | - | - | Vimentin (+), SMA (+) Partial expression of Hcaldesmon was observed. Focal or more extensive positivity for Syn was observed in three cases,  For cga (-) and NSE (-). AE1/AE3 (-), CD117 (-), LCA (-), S100 (-), and CD34 (-) | Endoscopic resection | - |
| 85 | X. Wang et al., 2019 (40) | 62 | M | healthy | Intermittent abdominal pain | Antrum | - | A hypoechoic region in the third or fourth layer of the gastric wall | Soft tissue shadow with smooth edges, visible punctate calcifications, and its wide base connected to the inner layer of the gastric antrum | SMA (+), vimentin (+), CD 34 (+), Factor VIII (+), Ki-67 <5% (+), ckp (-), CD 117 (-), and CD 31(-) | Laparoscopic wedge gastrectomy | Five months after the operation, a normal gastric mucosa was observed by gastroscopic examination. |
| 86 | G. Mavrogenis et al., 2019 (41) | 75 | F | - | Asymptomatic | Body | EUS: 1 | A hypoechoic lesion originating from the muscularis propria | - | SMA (+) vimentin (+) | Laparoscopic endoscopic cooperative surgery (LECS) | - |
| 87 | J. Hu et al., 2019(42) | 38 | M | - | Abdominal pain | Antrum | EUS: 1.9 | SMT, fourth layer Mild-hyperechoic round mass | - | - | Endoscopic submucosal dissection | Follow-up:3 |
| 88 | J. Hu et al., 2019 (42) | 62 | F | - | Abdominal discomfort | Body | EUS: 1 | SMT, fourth layer Mild-hyperechoic round mass | - | - | Endoscopic submucosal dissection | Follow-up:62 |
| 89 | J. Hu et al., 2019 (42) | 56 | F | - | Abdominal discomfort | Antrum | EUS: 1.1 | SMT, fourth layer Hypoechoic oval mass | - | - | Endoscopic submucosal dissection | Follow-up:48 |
| 90 | J. Hu et al., 2019 (42) | 52 | F | - | Abdominal pain | Antrum | EUS: 2.7 | SMT, fourth layer Mild-hyperechoic oval mass | - | - | Endoscopic full-thickness resection | Follow-up:60 |
| 91 | J. Hu et al., 2019 (42) | 59 | F | - | Abdominal discomfort | Antrum | EUS: 2.7 | SMT, fourth layer Mild-hyperechoic round mass | - | - | Laparoscopy | Follow-up:61 |
| 92 | J. Hu et al., 2019 (42) | 48 | F | - | Upper GI bleeding | Body | EUS: 3.5 | SMT with a ulcer on the surface, third, fourth layer Mild-hyperechoic oval mass With hypoechoic spots | - | - | Laparoscopy | Follow-up:18 |
| 93 | J. Hu et al., 2019 (42) | 47 | F | - | Abdominal discomfort | Antrum | EUS: 1.2 | SMT, fourth layer Mild-hyperechoic round mass | - | - | Laparoscopy | Follow-up:56 |
| 94 | J. Hu et al., 2019 (42) | 56 | M | - | Abdominal distention | Antrum | 2.8 | SMT, fourth layer Mild-hyperechoic oval mass | - | - | Laparoscopy | Follow-up:24 |
| 95 | J. Hu et al., 2019 (42) | 50 | M | - | Abdominal discomfort | Antrum | 2.7 | SMT | - | - | Laparoscopy | Follow-up:32 |
| 96 | J. Hu et al., 2019 (42) | 37 | F | - | Abdominal discomfort | Antrum | 2.2 | SMT, fourth layer  Mild-hyperechoic round mass | - | - | Laparoscopy | Follow-up:6 |
| 97 | J. Hu et al., 2019(42) | 46 | F | - | Abdominal discomfort | Antrum | 2 | SMT, fourth layer Mild-hyperechoic round mass | - | - | Laparoscopy | Follow-up:12 |
| 98 | H. Yoshida et al., 2019(43) | 60 | M | - | Incidental finding | Antrum | EUS: 2 | Hypoechoic tumor with heterogeneous echogenicity originating from the muscular propria layer | Gastric Submucosal tumor with a clear borderline and strong enhancement | SMA (+), chromogranin A (-), synaptophysin (-), c-Kit (-), DOG-1(-), CD34 (-), and desmin (-) | Laparoscopic partial gastrectomy | - |
| 99 | L. Toti et al., 2019(44) | 72 | M | liver neoplasms,moderate renal failure | Incidental finding | Greater curvature | Surgical: 6×4.5 CT: 6 | - | A dishomogeneous mass | SMA (+), synaptophysin (+), h-caldesmon (+), desmin (−), c-kit (−), DOG-1 (−), S-100 (−), CD34 (-), CK-pan (−) | Partial gastric resection | - |
| 100 | A. Farooq et al., 2019(45) | 75 | F | - | Incidental finding | Antrum | Surgical: 1.8 × 1.7 × 1.5 CT/EUS : 3.3 | A hypoechoic mass arising from the submucosa with internal necrosis | Exophytic mass | SMA(+), calponin (+) collagen type IV (+) , cytokeratin 18 (-), cytokeratin AE1/AE3(-) , synaptophysin (-), chromogranin (-), CD56 (-), CD117(c-kit) (-), DOG1 (-), GCDFP-15 (-), GATA3(-), mammoglobin (-), melanoma (-)cocktail (-), S100 (-) and CD45 (-) | Laparoscopic partial gastrectomy and partial omentectomy | - |
| 101 | A. Farooq et al., 2019(45) | 68 | M | - | Dysphagia | Fundus | Endoscopy: 1.25 × 1.25  EUS: 0.7 × 0.5 | An oval hypoechoic, homogenous intramural (subepithelial) lesion appeared to originate from the submucosa. | - | SMA (+) , vimentin (+), calponin (+), h-caldesmon (+) desmin (-), c-kit (-), CD34 (-), chromogranin(-), synaptophysin (-), cytokeratin(-), S100(-),and CD45 (-) | - | - |
| 102 | T. Namikawa et al.,2019(46) | 39 | M | healthy | discovered incidentally during a physical exam | Antrum | Endoscopy: 2 CT: 1.5 | Hypoechoic solid mass lesion with a small anechoic Component in the third or fourth tissues layer, suggesting a Lesion in the muscular layer of the stomach | A well -defined mass with strong homogeneous enhancement in the early | SMA (+), Desmin (-),  C-kit (-), CD34 (-), S-100 (-), chromogranin A (-), synaptophysin (-), And CD56 (-).  Approximately 1% of the cells were positive for the Proliferation marker Ki-67 | Laparoscopic distal gastrectomy with reginal lymph node dissection | - |
| 103 | S. Zhang et al., 2018(47) | 61 | M | - | Epigastric pain, nausea, and vomiting | Antrum | Surgical & CT: 1.7 EUS: 2.6 | Hypoechoic mass, with a clear border, located in the Gastric submucosa and muscularis propria layer | Solid, homogeneously enhanced mass. | Caldesmon (+) and (CD3 (+)), and desmin (-), pan-Cytokeratin (-), synaptophysin (-), human melanoma black 45 (-), CD34 (-), CD117 (-), and cytokeratin 18(-).  The Ki-67 labeling Index was 2% | Surgical partial gastrectomy | - |
| 104 | M. Wu et al., 2018(48) | 51 | F | healthy | Intermittent right abdominal pain | Antrum | Surgical: 1×1 CT: 1.3 | a thickening of the ileocecal wall, a slight narrowing of the intestine, and a 1.3 cm nodular enhancement lesion in the gastric antrum | A thickening of the ileocecal wall, a slight narrowing of the intestine, and enhancing lesion of the stomach | SMA (+), calponin (+), vimentin (+), syn (+), AE1/AE3 (-), cga (-) | Laparotomy surgical resection | At follow-up 3 months after surgery, the patient had no complaints. |
| 105 | D. Morte et al, 2018(49) | 41 | F | healthy | Upper GI bleeding | Antrum | CT:3×2.9×2.7 EUS: 3 | Well-circumscribed and homogeneous submucosal lesion | Hyperenhancing, prepyloric submucosal mass | Synaptophysin (+), SMA (+), CD 117 (-), and CD 34 (-) | Distal gastrectomy with Roux-en-Y Anastomosis | - |
| 106 | M. Masouminia et al., 2018(50) | 47 | F | - | Incidental finding | Antrum | EUS:2.5×2.3 | A well circumscribed submucosal heterogenous mass that appeared to be connected to muscularis propria | - | SMA (+), Synaptophysin (+), caldesmon (+), calponin (+), Cytokeratin AE1/AE3 (-), Desmin (-), chromogranin (-), S100 (-), C117/ckit (-), Dog-1(-) and CD34 (-) | Wedge gastrectomy | - |
| 107 | P. Yıldız et al., 2018(51) | 68 | M | healthy | Upper GI bleeding | Antrum | CT: 2.5×2.3  Surgical: 5.5×2.5×2.5  EUS: 4 | Hypervascularized mass, with microcalcifications inside, found in muscularis propria | Well-circumscribed, homogeneously enhanced solid submucosal tumor | Collagen type IV (focally +), SMA (+), vimentin (+), caldesmon (focally +), CD 117(-), DOG 1(-), S100 (-), CD34 (-), chromogranin (-), synaptophysin (-), CD56 (-), CD57 (-), PGP9.5 (-), desmin (-).Ki-67 index was 1% | Wedge resection and a partial omentectomy | - |
| 108 | J. Davis et al., 2018(52) | 46 | F | - | Incidental finding | Along the gastric wall | MRI: 1.4 | - | - | Synaptophysin (+), S100(-), CD45 (-), cytokeratin 7 (-), and chromo-granin (+) | Robot-assisted wedge excision | - |
| 109 | M. Ebi et al., 2017(53) | 45 | F | healthy | Incidental finding | Antrum | Endoscopy: 3 CT: 2.3 | Mosaic echo pattern and hypervascular mass in the muscular layer | Hypervascular in the arterial phase and exhibited continuous enhancement in the post-venous phase. | Α-SMA (+) , vimentin (+), collagen IV (+), synaptophysin (+), and caldesmon(+), desmin (-), cytokeratin (AE1/AE3) (-), CD56(-), chromogranin A (-), CD34(-), C-kit (-), and S-100 (-) Approximately 2–3% of the tumor cell nuclei were positive for the proliferation marker Ki-67 | Non-exposed endoscopic wall-inversion surgery (NEWS) | - |
| 110 | K. Duan et al., 2017(54) | 70 | F | - | Incidental finding | Lesser curvature | CT: 1.9 Gross: 2.2 | - | A lesion in the lesser curvature | SMS (+) | Wedge gastrectomy | - |
| 111 | T. Aoba et al., 2017(55) | 67 | F | healthy | Epigastric pain | Body | Surgical:  2 × 2 CT : 2 | Intraluminal hypoechoic tumor in the third or fourth layer of the stomach wall | A well-enhanced, intraluminal type tumor with no swollen lymph nodes | SMA(+) , vimentin (+), keratin (-), S-100 protein(-), C-kit (-), and CD34 (-) | Laparoscopy endoscopy cooperative surgery (LECS) | She had no recurrence and no complaints for 2 years after the operation. |
| 112 | T. Vig et al.,2017(56) | 49 | F | non-functioning right kidney with Grade-IV hydroureteronephrosis | Incidental finding | Antrum | US: 4×4 | - | - | SMA (+), Synaptophysin (-), Chromogranin (-), CD56 (-) Pancytokeratin (-) and CD117 (-) | Wedge resection | Patient is on regular follow-up and 24 months post surgery has had no recurrence or any metastatic event. |
| 113 | M. Oruç et al.,2016(57) | 38 | F | obesity,diabetes mellitus | Incidental finding | - | Surgical: 0.8 | - | - | S-100 (+); actin (+) and calponin (+); CD34 (+) chromogranin A (-), non-specific enolase (NSE (-), synaptophysin (-), CD56 (-), thyroglobulin (-), TTF1 (-), CD10 (-), CD117 (-), renal-cell carcinoma (RCC) (-), and panck (-). The ‎Ki‎-‎67 ‎index was approximately 1%. | Laparoscopic sleeve gastrectomy (LSG) | - |
| 114 | M. Chabowski et al., 2016 (58) | - | - |  | Syncope and gastrointestinal bleeding | Lesser curvature | - | - | - | - | Resection of the stomach with the tumor and end-to-end anastomosis according to the Rydygier’s method (Billroth I) using staplers. | - |
| 115 | C.C. Ruiz et al., 2016 (59) | 70 | F | - | Incidental finding | Antrum. | Surgical: 2.5×1.6×1.2 CT: 1.4 | - | the presence of a hyperdense lesion of about 14 mm, in the absence of lymphadenomegaly or metastatic disease. | SMA (+), desmin (-), chromogranin (-), synapthophisin (-), and keratin (-) | Gastric laparoscopic wedge resection | - |
| 116 | A. Papadelis et al., 2016(60) | 62 | F | healthy | Shortness of breath and persistent cough | Lesser curvature | EUS: 4.6×2.7cm | Mass adjacent to the angularis versus originating from the outside wall layers of the stomach pressing on the angularis | Enhancing exophytic mass from the stomach | SMA (+), calponin (+), pancytokeratin (Lu5) (-), chromogranin (-), synaptophysin (-), S100 protein (-), C117/c-kit (-) and CD34 (-) | Surgical excision | The patient recovered well postoperatively with an unremarkable follow-up. |
| 117 | Sh. Zaidi and M. Arafah et al., 2015(61) | 53 | F | anorexia and weight loss | Fullness and pain in the left hypochondrium | Fundus | Grossly 10 × 9 × 7.5 cm CT: 97 × 88 × 11 mm | - | A large, well-defined, cystic lesion, in close relation to the left lateral wall of the stomach. The lesion had a thick enhancing wall with thick peripheral internal septation | The proliferative index (Ki-67) in cellular areas was 15% SMA (+) , h-caldesmon (+) , vimentin (+) , collagen type IV (+) , and synaptophysin (+) CD117 (-), CD34 (-), cytokeratin (-), HMB-45(-), S-100 (-), desmin (-), and chromogranin(-) | Laparotomy and resection | The patient continues regular follow-up, and there was no evidence of recurrence 15 months after the resection of her gastric mass. |
| 118 | Handa et al.2015(62) | 24 | F | Healthy | vomit andmelena,tachycardia,and her hemoglobin level was extremely low (6.3 g/dL) | antral wall of the stomach | endoscopy:3 × 3 cm Surgical:3cm | a well-demarcated mass in the third and fourth layers of the gastric wall, with a hypoechoic pattern | a mass with dense homogeneous enhancement in the stomach wall. | vimentin (+), smooth muscle actin (+), and collagen type IV (+),CD34(-), synaptophysin(-), and chromogranin A(-) | laparoscopy-assisted surgical procedure:wedge resection | Ten months after the operation, the postoperative course remains uneventful without signs of relapse. |
| 119 | Casarotto et al. 2015(63) | 54 | M | - | intermittent epigastric pain and dyspepsia | Antrum. | endoscopy:1cm | the presence of a homogeneous hypoechoic mass arising from the muscularis propria | the presence of the mass and showed no evidence of metastasis | Vimentin (+)，Muscle-specific actin (+)，Calponin (+)，Caldesmon (+); Cytokeratin AE1/AE3(-)，S-100 (-)，CD34(-)，CD117(-)，Chromogranin(-)，Synaptophysin(-) | A partial gastrectomy with a Billroth II | After 36 months of follow-up the patient shows no signs of recurrence. |
| 120 | S.A Jain et al.,2014(64) | 40 | F | - | malena and hematemesis for 1 month. | the gastric pylorus | CT: 3.6 × 2.9 × 3.0 cm  Surgical:3 × 2.5 × 2 cm | - | a well-defined polypoidal soft tissue density lesion of size 36 × 29 × 30 mm seen within the first part of duodenum without exophytic component. Adjacent fat planes were clear. | SMA (+),CD34 (+,very focal);desmin(-), CD31(-), S-100(-), synaptophysin(-), chromogranin A(-), HMB 45(-), c-kit(-) and DOG-1(-) | distal gastrectomy with gastrojejunostomy | - |
| 121 | Wang et al. 2014(65) | 56 | M | - | Epigastric discomfort | Body | 3×2×1.5 | - | the tumors presented as masses located inside the gastric wall. | α-smooth muscle actin (ASMA)(+), laminin(+), collagen type IV(+), and vimentin (+) | Surgical resection | Follow-up:19months |
| 122 | Wang et al. 2014(65) | 55 | F | - | Upper GI bleeding | Antrum | 2.5×2.5×2 | - | the tumors presented as masses located inside the gastric wall. | α-smooth muscle actin (ASMA)(+), laminin(+), collagen type IV(+), and vimentin (+) | surgical resection | Follow-up:34m |
| 123 | Wang et al. 2014(65) | 50 | F | - | Epigastric discomfort | Antrum | 2.5×2×2 | - | the tumors presented as masses located inside the gastric wall | α-smooth muscle actin (ASMA)(+), laminin(+), collagen type IV(+), and vimentin (+) | surgical resection | Follow-up:43 |
| 124 | Wang et al. 2014(65) | 35 | F | - | Epigastric discomfort | Antrum | 2.7×2×2 | a solid mass originating from the superficial layer of the muscular propria. The hypoechoic lesion was prominent toward the cavity and had clear boundary | the tumors presented as masses located inside the gastric wall | α-smooth muscle actin (ASMA)(+), laminin(+), collagen type IV(+), and vimentin (+) | surgical resection | Follow-up:144 |
| 125 | Wang et al. 2014(65) | 47 | M | - | None | Body | 1.5×1.5×1 | - | the tumors presented as masses located inside the gastric wall | α-smooth muscle actin (ASMA)(+), laminin(+), collagen type IV(+), and vimentin (+) | surgical resection | Follow-up:1 |
| 126 | Wang et al. 2014(65) | 65 | M | - | Upper GI bleeding | Antrum | 2.3×2×2 | - | the tumors presented as masses located inside the gastric wall | α-smooth muscle actin (ASMA)(+), laminin(+), collagen type IV(+), and vimentin (+) | surgical resection | Follow-up:39 |
| 127 | Wang et al. 2014(65) | 64 | M | - | Diarrhea | Body | 8×6×5 | - | the tumors presented as masses located inside the gastric wall | α-smooth muscle actin (ASMA)(+), laminin(+), collagen type IV(+), and vimentin (+) | surgical resection | Follow-up:66 |
| 128 | Wang et al. 2014(65) | 43 | M | - | Epigastric discomfort | Antrum | 2.5×2×1.2 | - | the tumors presented as masses located inside the gastric wall | α-smooth muscle actin (ASMA)(+), laminin(+), collagen type IV(+), and vimentin (+) | surgical resection | Follow-up:55 |
| 129 | Wang et al. 2014(65) | 52 | F | - | Upper GI bleeding | Antrum | 2.0×2×1.5 | - | the tumors presented as masses located inside the gastric wall | α-smooth muscle actin (ASMA)(+), laminin(+), collagen type IV(+), and vimentin (+) | surgical resection | Follow-up:44 |
| 130 | Wang et al. 2014(65) | 62 | M | - | Diarrhea | Body | 1.5×1.5×1 | - | the tumors presented as masses located inside the gastric wall | α-smooth muscle actin (ASMA)(+), laminin(+), collagen type IV(+), and vimentin (+) | surgical resection | Follow-up:60 |
| 131 | Wang et al. 2014(65) | 45 | M | - | None | Antrum | 2.2×2×2 | - | the tumors presented as masses located inside the gastric wall | α-smooth muscle actin (ASMA)(+), laminin(+), collagen type IV(+), and vimentin (+) | surgical resection | Follow-up:74 |
| 132 | Orellana et al. 2011(66) | 26 | F | - | a year of intermittent epigastric pain but no other symptoms | the anterior wall of the gastric body | Surgical:2cm | a hypoechoic lesion arising from the muscularis propria, without deep involvement, compatible with a gastrointestinal stromal tumor (GIST). | - | SMA (+) | partial gastrectomy | - |
| 133 | HQ.Fang et al.2010(67) | 60 | F | - | a gastric mass found at upper gastrointestinal endoscopy | Antrum | endoscopy:1.5×1.5 EUS：1.5×1.2 surgical:1×1cm | a sharply demarcated homogeneous hypoechoic mass measuring 1.5 cm × 1.2 cm in the third and fourth sonographic layers of gastric wall with rich blood supply | - | vimentin (+), SMA (+) and actin (+);S-100(-), CD34(-), CD117(-), Desmin(-), CD56(-), synaptophysin(-), chromogranin A(-), neurone specific enolase(-) and cytokeratin(-) | wedge resection of the tumor. | - |
| 134 | K C Chou et al.2010 (68) | 54 | F | chronic renal failure | an episode of tarry stool | Antrum | EUS：3cm | a 3-cm, well-defined, hyperechoic tumor with a calcified spot | an hyperintense, homogeneously enhancing tumor | - | - | - |
| 135 | Chuang-Chi Huang et al. 2010(69) | 37 | F | - | intermittent epigastric discomfort over a 2-month period | Antrum | surgical:2.0 × 1.5 cm | the tumor was within the muscularis propria layer and had a heterogeneous appearance | a hypervascular submucosal tumor at the posterior, medial and inferior wall of the antrum, near the pylorus ring | smooth muscle actin(+), cytokeratin(-) and CD56(-) | wedge resection. | The patient recovered uneventfully and was discharged 5 days after surgery. |
| 136 | Vassiliou et al. 2010(70) | 72 | F | - | an episode of melena | Antrum | surgical:2 × 2 × 1.7 cm | the submucosal lesion which originated from the muscularis propria, measured 1.9 × 2.4 cm and was extending in the second, third and fourth layer of the stomach. | a 3 cm localized, prepyloric enhancing mass at the lesser curvature of the stomach | smooth muscle actin(+),vimentin(+);desmin(-), CD34(-), CD117(-), S-100 (-)and cytokeratins (AE1/3, CAM 5,2)(-). The proliferating marker Ki-67 was < 5%. | antrectomy and Roux-en-Y anastomosis | The patient recovered uneventfully and was discharged 5 days after surgery. |

References:

1. Song DH, Kim TH, An HJ. Gastric Glomus Tumor with Neuroendocrine Features: A Diagnostic Pitfall for Neuroendocrine Tumors. Diagnostics (Basel, Switzerland). 2025;15(22).

2. Yang X, Guo Y, Yan X, Xu B, Cui Z, Guo Z. Case report: One case of precise resection of gastric glomus tumor by gastroscopy combined with laparoscopy. Frontiers in oncology. 2024;14:1501442.

3. Nimura Y, Nishikawa T, Nagura A, Kurobe T, Yashika J, Ko R, et al. A Gastric Glomus Tumor Diagnosed Preoperatively Using Contrast-enhanced Harmonic Endoscopic Ultrasonography and Endoscopic Ultrasonography-guided Fine Needle Aspiration. Internal medicine (Tokyo, Japan). 2025.

4. Xu S, Xu T, Zhi Y, Dong F, Wu C, Zheng M. Malignant gastric glomus tumor with heterochronous liver metastases: a case report and review of the literature. Journal of medical case reports. 2025;19(1):199.

5. Toyomasu Y, Nakazato K, Shitara Y, Ishizaki M, Saeki H, Shirabe K. A case of gastric glomus tumor with metachronous liver metastasis after laparoscopic partial gastrectomy. International journal of surgery case reports. 2025;131:111357.

6. Fejes R, Gyorgyev KS, Góg C, Krenács L, Zombori T, Széll ZE, et al. Gastric glomus tumor with uncertain malignant potential: case report of a rare cause of upper gastrointestinal bleeding. World journal of surgical oncology. 2024;22(1):299.

7. Thalji MM, Alnajjar YA, Mohammad M, Khadra H, Bannoura S, Al-Ashhab H. Gastric glomus tumor with a rare presentation: a case report and review of the literature. Annals of medicine and surgery (2012). 2024;86(12):7356-61.

8. Huang J, Yuan C, Zhang S, Qu T, Suo J. A case of malignant gastric glomus tumor and literature review: A case report. Medicine. 2024;103(32):e39208.

9. Bronze S, Botto I, Ruivo L, Oliveira T, Moura M, Ribeiro LC, et al. Gastric Glomus Tumor Resected by Submucosal Tunneling Endoscopic Resection. ACG case reports journal. 2024;11(11):e01553.

10. Cláudio JC, Filizzola PAM, Figueiredo HF, Lira DL, da Costa AP, Cardoso TM. Endoscopy-assisted laparoscopic wedge-resection of gastric glomus tumor: A case report. International journal of surgery case reports. 2024;122:110100.

11. Deacu M, Bosoteanu M, Orășanu CI, Ursica OA, Voda RI. A 65-Year-Old Man Presenting to the Emergency Department with Gastric Hemorrhage Caused by a Glomus Tumor. The American journal of case reports. 2024;25:e942610.

12. Yu F, Ma J, Huang D, Guan B. Gastric glomus tumor of uncertain malignant potential. Asian journal of surgery. 2023;46(2):1126-7.

13. Alkhateb O, Daaboul O, Daaboul B, Abouharb R, Alazmeh MA, Hamed G. Glomangiomyoma of the Stomach: Case Report. Case reports in gastroenterology. 2023;17(1):185-90.

14. Deng M, Luo R, Huang J, Luo Y, Song Q, Liang H, et al. Clinicopathologic features of gastric glomus tumor: A report of 15 cases and literature review. Pathology oncology research : POR. 2022;28:1610824.

15. Malik A, Yousaf MN, Samiullah S, Tahan V. Gastric Glomus Tumors: The Roles of Endoscopic Ultrasound and Shared Decision-Making. Case reports in gastroenterology. 2023;17(1):356-61.

16. Mohamed WT, Jahagirdar V, Jaber F, Ahmed M, Fatima I, Chhabra R, et al. Glomus Tumor of the Stomach Presenting With Upper Gastrointestinal Bleeding: A Case Report. Journal of investigative medicine high impact case reports. 2023;11:23247096231192891.

17. Ezeh KJ, Boateng W, Paudel B, Ezeudemba O, Botros Y. A Case of Gastric Glomus Tumor Misdiagnosed as Carcinoid Tumor. Cureus. 2023;15(1):e34316.

18. Frosio F, Petruzziello C, Poiasina E, Pisano M, Lucianetti A. Locally Advanced Glomus Tumor of the Stomach With Synchronous Liver Metastases: Case Report and Literature Review. Cureus. 2023;15(12):e51041.

19. Ayash A, Elkomy N, Al-Mohannadi MJ, Al Kaabi SR, Petkar M. Gastric glomus tumor presenting with massive upper GI bleeding: A challenging to diagnose and treat tumor. Clinical case reports. 2022;10(8):e6172.

20. Ezeh KJ, Rajwana Y, Paudel B, Shen T, Botros Y. Gastric Glomus Tumor Presenting With Gastrointestinal Bleed and Pulmonary Embolism: A Rare Entity With Management Dilemma. Cureus. 2022;14(6):e25632.

21. Mehmood F, Jamil H, Khalid A. Gastric Glomus Tumor: A Rare Cause of Acute Blood Loss Anemia. Cureus. 2022;14(4):e24511.

22. Osama MA, Khetan D, Dhawan S, Badwal S. Glomus Tumor of Scrotum and Stomach: Usual Tumor at Unusual Locations. Indian journal of surgical oncology. 2022;13(2):235-8.

23. Bai B, Mao CS, Li Z, Kuang SL. Endoscopic ultrasonography diagnosis of gastric glomus tumors. World journal of clinical cases. 2021;9(33):10126-33.

24. Lee K, Ahn B, Hong SM, Ahn JY, Gong CS, Ryu JS. A Case of Glomus Tumor Mimicking Neuroendocrine Tumor on (68) Ga-DOTATOC PET/CT. Nuclear medicine and molecular imaging. 2021;55(6):315-9.

25. Brotherton T, Khneizer G, Nwankwo E, Yasin I, Giacaman M. Gastric Glomus Tumor Diagnosed by Upper Endoscopy. Cureus. 2021;13(12):e20703.

26. Tantia M, Suryawanshi PR, Gupta A, Rachakatla P. Gastric glomus tumour: A case report. Journal of minimal access surgery. 2021;17(4):551-3.

27. Vyawahare MA, Musthyala BN, Tayade RT. Gastric glomus tumor: A rare etiology of upper gastrointestinal bleed. Indian journal of pathology & microbiology. 2021;64(4):795-8.

28. Wang WH, Shen TT, Gao ZX, Zhang X, Zhai ZH, Li YL. Combined laparoscopic-endoscopic approach for gastric glomus tumor: A case report. World journal of clinical cases. 2021;9(24):7181-8.

29. Mendo R, Barosa R, Pinto Marques P, Albuquerque C, Santos CM. An Unusual Gastric Subepithelial Lesion: Expect the Not so Expectable. GE Portuguese journal of gastroenterology. 2022;29(1):68-70.

30. Sethi S, Verma AK, Jain N, Goel V, Puri SK. Multimodality imaging of gastric glomus tumor presenting with upper GI bleed. Tropical gastroenterology : official journal of the Digestive Diseases Foundation. 2016;37(2):139-41.

31. Alsahwan AG, Alfaraj ZM, AlSafwani J, Bunaiyan AH, AlKhalifah RH, Al-Saba'a SA, et al. Rare gastric neoplasm: Malignant glomus tumor of the stomach. A case report. International journal of surgery case reports. 2021;81:105802.

32. Tsagkataki ES, Flamourakis ME, Gkionis IG, Giakoumakis MI, Delimpaltadakis GN, Kazamias GM, et al. Gastric glomus tumor: a case report and review of the literature. Journal of medical case reports. 2021;15(1):415.

33. Singh S, Kumar A, Singh V. Gastric Glomus Tumor. Nigerian journal of surgery : official publication of the Nigerian Surgical Research Society. 2020;26(2):162-5.

34. Hansen T, Titze U, Trachte F, Maschuw K, Hiller W, Tebbe JJ. [Uncommon tumor of the gastric wall]. Der Pathologe. 2021;42(3):328-32.

35. Mago S, Pasumarthi A, Miller DR, Saade R, Tadros M. The Two Challenges in Management of Gastric Glomus Tumors. Cureus. 2020;12(7):e9251.

36. Hasuda H, Hu Q, Miyashita Y, Zaitsu Y, Tsuda Y, Hisamatsu Y, et al. Gastric glomus tumor with a preoperative diagnosis by endoscopic ultrasonography-guided fine needle aspiration: a case report. International cancer conference journal. 2021;10(1):35-40.

37. Rossi UG, Rutigliani M, Paparo F, Filauro M. Gastric glomus tumor: Endoscopy, MD-CT and pathologic features. Gastroenterologia y hepatologia. 2021;44(1):35-6.

38. Vieites Branco I, Silva JC, Pinto F, Pires F, Almeida A. Rare mesenchymal antral gastric tumors: Case reports of glomus tumor and plexiform fibromyxoma. Radiology case reports. 2020;15(1):71-6.

39. Lin J, Shen J, Yue H, Li Q, Cheng Y, Zhou M. Gastric Glomus Tumor: A Clinicopathologic and Immunohistochemical Study of 21 Cases. BioMed research international. 2020;2020:5637893.

40. Wang X, Hanif S, Wang B, Chai C. Management of gastric glomus tumor: A case report. Medicine. 2019;98(38):e16980.

41. Mavrogenis G, Mitropapas G, Kyriakidou V, Bazerbachi F. Laparoscopic endoscopic cooperative surgery for a gastric glomus tumor previously diagnosed by endoscopic ultrasound fine-needle biopsy. Annals of gastroenterology. 2020;33(2):219.

42. Hu J, Ge N, Wang S, Liu X, Guo J, Wang G, et al. The Role of Endoscopic Ultrasound and Endoscopic Resection for Gastric Glomus: A Case Series and Literature Review. Journal of translational internal medicine. 2019;7(4):149-54.

43. Yoshida H, Asada M, Marusawa H. Gastrointestinal: Glomus tumor: A rare submucosal tumor of the stomach. Journal of gastroenterology and hepatology. 2019;34(5):815.

44. Toti L, Manzia TM, Roma S, Meucci R, Blasi F, Ferlosio A, et al. Rare malignant glomus tumor of the stomach with liver metastases. Radiology case reports. 2019;14(4):463-7.

45. Farooq A, Goyal A, Giorgadze T, Scherr G, Evans JJ, Hartley CP. Cytomorphological features of glomus tumors arising in the stomach: A series of two cases diagnosed on FNA. Annals of diagnostic pathology. 2019;42:42-7.

46. Namikawa T, Tsuda S, Fujisawa K, Iwabu J, Uemura S, Tsujii S, et al. Glomus tumor of the stomach treated by laparoscopic distal gastrectomy: A case report. Oncology letters. 2019;17(1):514-7.

47. Zhang S, Zhang J, Wang C. Glomus Tumor of the Stomach-A Tumor That Needs to Be Differentiated From Gastrointestinal Stromal Tumor. Clinical gastroenterology and hepatology : the official clinical practice journal of the American Gastroenterological Association. 2018;16(3):A29-a30.

48. Wu M, Zhou T, Cao D, Qu L, Cao X. Glomus tumor of the stomach: A case report. Medicine. 2018;97(45):e13132.

49. Morte D, Bingham J, Sohn V. Gastric Glomus Tumor: An Uncommon Source for an Acute Upper GI Bleed. Case reports in gastrointestinal medicine. 2018;2018:7961981.

50. Masouminia M, Ghani HA, Foote D, Hari D, French S. Rare presentation of the glomus tumor in the stomach. Experimental and molecular pathology. 2018;104(1):9-11.

51. Yıldız P, Gücin Z, Arıcı DS, Malya F, Baysal B. Glomus tumor of the stomach. Turkish journal of surgery. 2018;34(1):62-4.

52. Davis J, Petterson M, Newell J, Lauwers GY, Royce T, Demeure MJ. Micrometastatic gastric glomus tumour confirmed by next-generation sequencing. Histopathology. 2018;72(2):351-4.

53. Ebi M, Sugiyama T, Yamamoto K, Saito T, Inoue T, Yamaguchi Y, et al. A gastric glomus tumor resected using non-exposed endoscopic wall-inversion surgery. Clinical journal of gastroenterology. 2017;10(6):508-13.

54. Duan K, Chetty R. Gastric glomus tumor: clinical conundrums and potential mimic of gastrointestinal stromal tumor (GIST). International journal of clinical and experimental pathology. 2017;10(7):7905-12.

55. Aoba T, Kato T, Hiramatsu K, Shibata Y, Yoshihara M, Yamaguchi N, et al. A case of gastric glomus tumor resection using laparoscopy endoscopy cooperative surgery (LECS). International journal of surgery case reports. 2018;42:204-7.

56. Vig T, Bindra MS, Kumar RM, Alexander S. Gastric Glomus Tumour Misdiagnosed as Gastric Carcinoid: An Unfamiliar Entity with Aids to Diagnosis and Review of Literature. Journal of clinical and diagnostic research : JCDR. 2017;11(5):Ed32-ed3.

57. Oruç MT, Çakir T, Aslaner A, Çekiç S, Sakar A, Yardimci EC. Incidental gastric glomus tumor after laparoscopic sleeve gastrectomy. Autopsy & case reports. 2016;6(1):47-50.

58. Chabowski M, Paszkowski A, Skotarczak J, Dorobisz T, Leśniak M, Janczak D, et al. Glomus Tumor of the Stomach - A Case Report and A Literature Review. Polski przeglad chirurgiczny. 2016;88(6):356-8.

59. Castro Ruiz C, Carlinfante G, Zizzo M, Giunta A, Ronzoni R, Azzolini F, et al. Glomus Tumor of the Stomach: GI Image. Journal of gastrointestinal surgery : official journal of the Society for Surgery of the Alimentary Tract. 2017;21(6):1099-101.

60. Papadelis A, Brooks CJ, Albaran RG. Gastric glomus tumor. Journal of surgical case reports. 2016;2016(11).

61. Zaidi S, Arafah M. Malignant Gastric Glomus Tumor: A Case Report and Literature Review of a Rare Entity. Oman medical journal. 2016;31(1):60-4.

62. Handa Y, Kano M, Kaneko M, Hirabayashi N. Gastric Glomus Tumor: A Rare Cause of Upper Gastrointestinal Bleeding. Case reports in surgery. 2015;2015:193684.

63. Casarotto A, Zarantonello FR, Piccirillo G, Criscenti P, Verza M, Zirillo M, et al. Gastric glomus tumor: a rare case of dyspepsia. Endoscopy. 2015;47 Suppl 1 UCTN:E75-6.

64. Jain SA, Agarwal L, Goyal A, Kumar R, Nadkarni S, Ameta A, et al. Gastric glomus tumor. Journal of surgical case reports. 2014;2014(6).

65. Wang ZB, Yuan J, Shi HY. Features of gastric glomus tumor: a clinicopathologic, immunohistochemical and molecular retrospective study. International journal of clinical and experimental pathology. 2014;7(4):1438-48.

66. Orellana F, Onetto C, Balbontín P, Videla D, Manriquez L, Plass R, et al. Gastric glomus tumor: report of one case and review. Endoscopy. 2011;43 Suppl 2 UCTN:E71-2.

67. Fang HQ, Yang J, Zhang FF, Cui Y, Han AJ. Clinicopathological features of gastric glomus tumor. World journal of gastroenterology. 2010;16(36):4616-20.

68. Chou KC, Yang CW, Yen HH. Rare gastric glomus tumor causing upper gastrointestinal bleeding, with review of the endoscopic ultrasound features. Endoscopy. 2010;42 Suppl 2:E58-9.

69. Huang CC, Yu FJ, Jan CM, Yang SF, Kuo YT, Hsieh JS, et al. Gastric glomus tumor: a case report and review of the literature. The Kaohsiung journal of medical sciences. 2010;26(6):321-6.

70. Vassiliou I, Tympa A, Theodosopoulos T, Dafnios N, Fragulidis G, Koureas A, et al. Gastric glomus tumor: a case report. World journal of surgical oncology. 2010;8:19.
