# Supplementary material for: A Case Report: Gastric glomus tumor with RAD50 mutation and therapeutic advances
Source: Front Oncol. 2026 Mar 4;16:1745685. doi: 10.3389/fonc.2026.1745685 (PMC12995647; doi:10.3389/fonc.2026.1745685)
Supplement: Supplementary file 2 [file Table2.docx]

**CARE Checklist of information to include when writing a case report**

| Topic | Item | Checklist item description | Reported on Line |
| --- | --- | --- | --- |
| Title | 1 | The diagnosis or intervention of primary focus followed by the words “case report” | Lines1-2 |
| Key Words | 2 | 2 to 5 key words that identify diagnoses or interventions in this case report, including "case report" | Lines11-12 |
| Abstract (no references) | 3a | Introduction: What is unique about this case and what does it add to the scientific literature? | Lines16-18 |
|  | 3b | Main symptoms and/or important clinical findings | Lines18-21 |
|  | 3c | The main diagnoses, therapeutic interventions, and outcomes | Lines20-24 |
|  | 3d | Conclusion—What is the main “take-away” lesson(s) from this case? | Lines25-27 |
| Introduction | 4 | One or two paragraphs summarizing why this case is unique (may include references | Lines38-84 |
| Patient Information | 5a | De-identified patient specific information | Lines86 |
|  | 5b | Primary concerns and symptoms of the patient | Lines86-90 |
|  | 5c | Medical, family, and psycho-social history including relevant genetic information | Lines90-97 |
|  | 5d | Relevant past interventions with outcomes | Lines114-118 |
| Clinical Findings | 6 | Describe significant physical examination (PE) and important clinical findings | Lines96-101 |
| Timeline | 7 | Historical and current information from this episode of care organized as a timeline | Figure 5 |
| Diagnostic Assessment | 8a | Diagnostic testing (such as PE, laboratory testing, imaging, surveys) | Lines102-113 |
|  | 8b | Diagnostic challenges (such as access to testing, financial, or cultural) | Lines105-108 |
|  | 8c | Diagnosis (including other diagnoses considered) | Lines104-113 |
|  | 8d | Prognosis (such as staging in oncology) where applicable | Lines113-114 |
| Therapeutic Intervention | 9a | Types of therapeutic intervention (such as pharmacologic, surgical, preventive, self-care) | Lines127-131;  133-136;  150-154;  158-174. |
|  | 9b | Administration of therapeutic intervention (such as dosage, strength, duration) | Lines132-133;  150-154;158-161;165-167;170-172. |
|  | 9c | Changes in therapeutic intervention (with rationale) | Lines136-140,141-154;158-174 |
| Follow-up and Outcomes | 10a | Clinician and patient-assessed outcomes (if available) | - |
|  | 10b | Important follow-up diagnostic and other test results | - |
|  | 10c | Intervention adherence and tolerability (How was this assessed?) | Lines179-182 |
|  | 10d | Adverse and unanticipated events | - |
| Discussion | 11a | A scientific discussion of the strengths AND limitations associated with this case report | Lines191-263 |
|  | 11b | Discussion of the relevant medical literature with references | Lines184-190 |
|  | 11c | The scientific rationale for any conclusions (including assessment of possible causes) | Lines198-200;220-222;240-242 |
|  | 11d | The primary “take-away” lessons of this case report (without references) in a one paragraph conclusion | Lines265-284 |
| Patient Perspective | 12 | The patient should share their perspective in one to two paragraphs on the treatment(s) they received | Lines179-182;232-240 |
| Informed Consent | 13 | Did the patient give informed consent? Please provide if requested | √ Yes ☐ No |
